# A novel hybrid NSGA-III and machine learning framework for modeling wheat yield variability using climatic, edaphic, and nutritional drivers

**DOI:** 10.1038/s41598-026-48918-0

**Published:** 2026-05-06

**Authors:** Mohsen Jahan, Mohammad Bannayan, Mehdi Nassiri-Mahallati, Fatemeh Yaghoubi

**Affiliations:** Department of Agrotechnology, Faculty of Agriculture, Ferdowsi University of Mashhad (FUM), P.O. Box 9177948978, Mashhad, Iran

**Keywords:** Agroclimatic variable, Arid climate, Machine learning, Precipitation, Soil salinity, Climate sciences, Environmental sciences, Mathematics and computing, Plant sciences

## Abstract

Accurate prediction of crop yield remains a critical research priority due to the increasing vulnerability of agricultural systems to climate change and the growing need for food security. In this study, we developed a hybrid modeling framework to predict irrigated wheat yield in Razavi Khorasan Province, Iran, using long-term (2004–2023) climatic, edaphic, and nutritional datasets comprising 47 variables collected across 17 counties. After preprocessing, feature selection was performed using an integrated approach combining Mutual Information (MI), Recursive Feature Elimination (RFE), and the advanced NSGA-III multi-objective optimization algorithm. Final yield prediction was conducted with a Stacking Regressor meta-learner incorporating LightGBM (LGBM) and a Deep Neural Network (DNN). The optimal subset of 10 features—Tmin, TS, K, Silt, EC, HCO₃, Mg, Prec_OC, AI_Clay, and a regional indicator variable (county_te) —achieved a test-set R^2^ of 0.44, reflecting a moderate yet meaningful level of explained variance given the multidimensional, nonlinear, and environmentally heterogeneous nature of the wheat production system. SHAP (SHapley Additive Explanations) analysis further highlighted the dominant influence of regional heterogeneity alongside complex interactions among climatic, soil, and nutritional factors. While the model does not capture all sources of variability, the results demonstrate that this hybrid optimization–learning pipeline reliably characterizes a substantial portion of wheat yield variation and offers a practical decision-support tool for site-specific management and climate adaptation planning.

**Supplementary Information:**

The online version contains supplementary material available at 10.1038/s41598-026-48918-0.

## Introduction

Agricultural systems worldwide are increasingly vulnerable to climate change and extreme weather events, threatening food security and socio-economic stability^1^. This vulnerability, coupled with unprecedented global population growth (projected at 9.7 billion by 2050), declining water and soil resources, and intensifying climate change, places dual pressure on food production systems, making food security one of the most critical challenges of the twenty-first century^2,3^. Under these conditions, merely increasing production is insufficient,optimizing input use, improving productivity, reducing waste, and accurately predicting crop yields are also fundamental priorities for policymakers and researchers^4^. Yield fluctuations vary spatiotemporally, and these differences stem from variations in inputs, farming practices, soil, and climatic conditions^5^. Climate change profoundly impacts crop yields, with simple indicators such as temperature and precipitation alone explaining about 30–40% of the variance in annual yield fluctuations^6^ (Lobell and Field, 2007). Specifically, some studies have shown that, globally, climate change explains about one-third (28–39%) of observed yield variability (Ray et al., 2015)^7,8^. Future climate models also predict and confirm decreases in crop yields, with production declining significantly due to warming and shorter growth periods^9,10^. The occurrence and recurrence of extreme events such as prolonged droughts, floods, and frost, particularly in arid and semi-arid regions, pose numerous challenges to crop production, potentially rapidly undermining agricultural infrastructure^11,12^. In these regions, farmers strive to cope with annual climate variations, and adaptation strategies such as adjusting planting dates and optimizing irrigation regimes help mitigate the negative effects of drought and heat stress^13,14^.

Wheat (Triticum aestivum L.) is the world’s most important cereal and a staple crop in many arid and semi-arid regions, constituting a major pillar of food supply with a share of about 21% of global calorie production^15^ (Liu et al., 2021)^8^. This crop is particularly sensitive to climate fluctuations. Global demand for wheat is estimated to increase by up to 60% by 2050, while climate variability intensifies the declining trend in its annual yield^15^. The negative impact of rising temperatures on the yield of wheat, barley, and corn has been demonstrated globally^9^. The area of farmland in arid and semi-arid regions constitutes about 40% of the world’s total farmland area and is still increasing (Liu et al., 2021)^8^. The spatiotemporal variability of wheat yields globally and in Iran results not only from climate change but also from differences in access to water resources, crop management, and farming equipment^16–18^. For example, in a panel study in North Khorasan province, over 63% of wheat yield variability was explained by climatic variables^16^. Similarly, in the eastern United States, 36% of yield variation was attributed to weather patterns, emphasizing the effectiveness of temperature and SPEI indicators in identifying climate-sensitive areas^17,18^.

In Iran, numerous studies have reported decreases in seasonal precipitation, especially in spring and autumn, which exacerbates water scarcity and heat stress. Modeling the impact of climate change on the country’s cropping systems in recent years has indicated a decreasing trend in wheat yield, and predictions of up to a 48% yield reduction in some regions by 2021 have been realized^19^. Similar approaches have been successfully applied to other strategic crops in Iran. For instance, Eskandari et al.^20^ used soil properties—including salinity, exchangeable sodium percentage, gypsum, lime, gravel, available potassium, and phosphorus—to predict date palm yield across five date-producing provinces using multivariate regression and artificial neural networks. Their results showed that regression models, despite their simplicity, provided predictions close to observed yields and were preferred for practical applications^20^. Building on similar soil-centric approaches, Seyedmohammadi et al.^21^ applied multiple predictive models—including multivariate linear and nonlinear regression, ANFIS, FFBP-ANN, and Random Forest—to estimate pistachio yield across five Iranian provinces using 124 orchards. Their Random Forest model achieved the highest accuracy (R^2^ = 0.96) and identified gravel, electrical conductivity (EC), exchangeable sodium, CaCO₃, gypsum, and available phosphorus and potassium as the most influential soil factors^21^. A follow-up hybrid SVM-Firefly model further improved pistachio yield prediction to R^2^ = 0.94 using the same soil predictors, highlighting the gain from meta-heuristic optimization^22^. The same research group also applied Fuzzy TOPSIS with soil constraints (depth, EC, ESP, CaCO₃, gypsum) to prioritize maize, rapeseed, and soybean cultivation in Ardabil, ranking maize highest^23^. Later, they developed an AHP-Matter Element hybrid with GIS to map barley suitability in Ardabil using identical soil constraints, achieving R^2^ = 0.947 with observed yield^24^.

Accurate prediction of crop yields, especially wheat, helps farmers employ resources such as water, fertilizer, and labor more effectively. These predictions also aid in marketing and distribution planning, allowing stakeholders to prepare for supply and demand fluctuations, reduce waste, and ensure food availability^2^. Razavi Khorasan province in northeastern Iran, due to its strong spatial climatic heterogeneity—from semi-arid plains to intermontane semi-arid plains—provides a valuable testing ground for developing such predictive approaches^16^. As the relationships between climatic, edaphic, nutritional, and management variables become more complex, the need for advanced methods to model and predict wheat yield increases^25,26^.

Although process-based models with extensive capabilities are available today, hyperparameter tuning, calibration, and validation under specific conditions remain challenging, and output errors persist^25,26^. Machine learning (ML) algorithms have therefore emerged as valuable tools for real-time yield prediction (Benos et al., 2021). ML, with its high capacity to identify nonlinear and multivariate patterns, has gained popularity in recent years^4,27,28^. Numerous studies report that ML-based models outperform classical statistical models, such as multiple linear regression, in predicting crop yields^29,30^. For example, the root mean square error of a Random Forest model in a regional study was 163.90 kg/ha (winter wheat yield), compared to 653.39 kg/ha for a linear regression model, demonstrating higher accuracy^3^.

Novel methods like self-training Random Forest, which expand labeled datasets to improve prediction accuracy, show that model performance can increase without extensive initial data^31^. Recent research emphasizes tuning hyperparameters via grid search and cross-validation, leading to significant improvements in ML model accuracy^27^. Random Forest has been widely applied in agriculture due to its ability to model nonlinear relationships, resist overfitting, and provide variable importance indicators^18,32^. Compared to traditional regression models, ML-clustering frameworks enable nonlinear and context-specific modeling under diverse climatic regimes^27,28^. SHAP analysis of the CatBoost model revealed that Tmin, Tave, and SD significantly influence fresh corn water demand, and interactions between sunshine hours and rising temperatures increase water requirements^33^. Van Klompenburg et al.^34^ emphasized the need for region-specific ML frameworks, a gap addressed by subsequent studies^34,35^.

Recent studies highlight the importance of evaluating multiple modeling techniques in parallel; for instance, Khashei-Siuki et al.^36^ compared Multilayer Perceptron (MLP) and Adaptive Neuro-Fuzzy Inference Systems (ANFIS) for wheat yield prediction under dry conditions, with ANFIS outperforming MLP during testing. Other ML applications have used agro-morphological features and dimensionality reduction techniques like PCA; Parsaeian et al.^37^ demonstrated that Gaussian Process Regression (R^2^ = 0.99) and RBF neural networks (R^2^ = 0.91) accurately estimated sesame yield. ML, paired with interpretable techniques like SHAP, provides predictions and explanations that support targeted policies for sustainable intensification^38,39^. While some previous research applied clustering for yield prediction^37,40^, none integrated it with SHAP analysis to decode drivers across heterogeneous agro-climatic regions, an innovation critical for adaptive management.

The NSGA-III algorithm, evolved from NSGA-II^41^, uses a reference direction system to maintain solution diversity in multidimensional search spaces and is particularly suitable for optimization problems with numerous objectives, including agricultural systems with multiple performance indicators^41,42^. NSGA-III optimizes hyperparameters such as learning rates or tree depth in models like XGBoost, balancing accuracy and training time^43^. Its capacity to handle complex nonlinear relationships makes it suitable for agricultural datasets with intricate patterns^43^. Since its introduction, NSGA-III has been applied in diverse fields including precision agriculture, multi-objective engineering, and natural resource management^44^. For example, combining a yield prediction model with NSGA-III enabled optimization of crop portfolios based on economic and environmental indicators^44^. Using NSGA-III with the AquaCrop growth simulation model also optimized irrigation strategies for winter wheat^45^. Evolved versions like θ-NSGA-III and A-NSGA-III further improve efficiency^46^.

Given the lack of literature on applying NSGA-III for strategic crops like wheat, this study combines NSGA-III with LGBM and a deep neural network in a hybrid metalearner pipeline to optimize feature selection affecting wheat grain yield using climatic, edaphic, and nutritional features. This approach integrates feature engineering, multi-objective optimization, and ML methods to better understand ecophysiological relationships and improve input management under climate variability. The study examines key drivers, such as minimum temperature and edaphic-nutritional features, using SHAP analysis.

## Materials and methods

### Study area, data collection and preprocessing

**Fig. 1:**
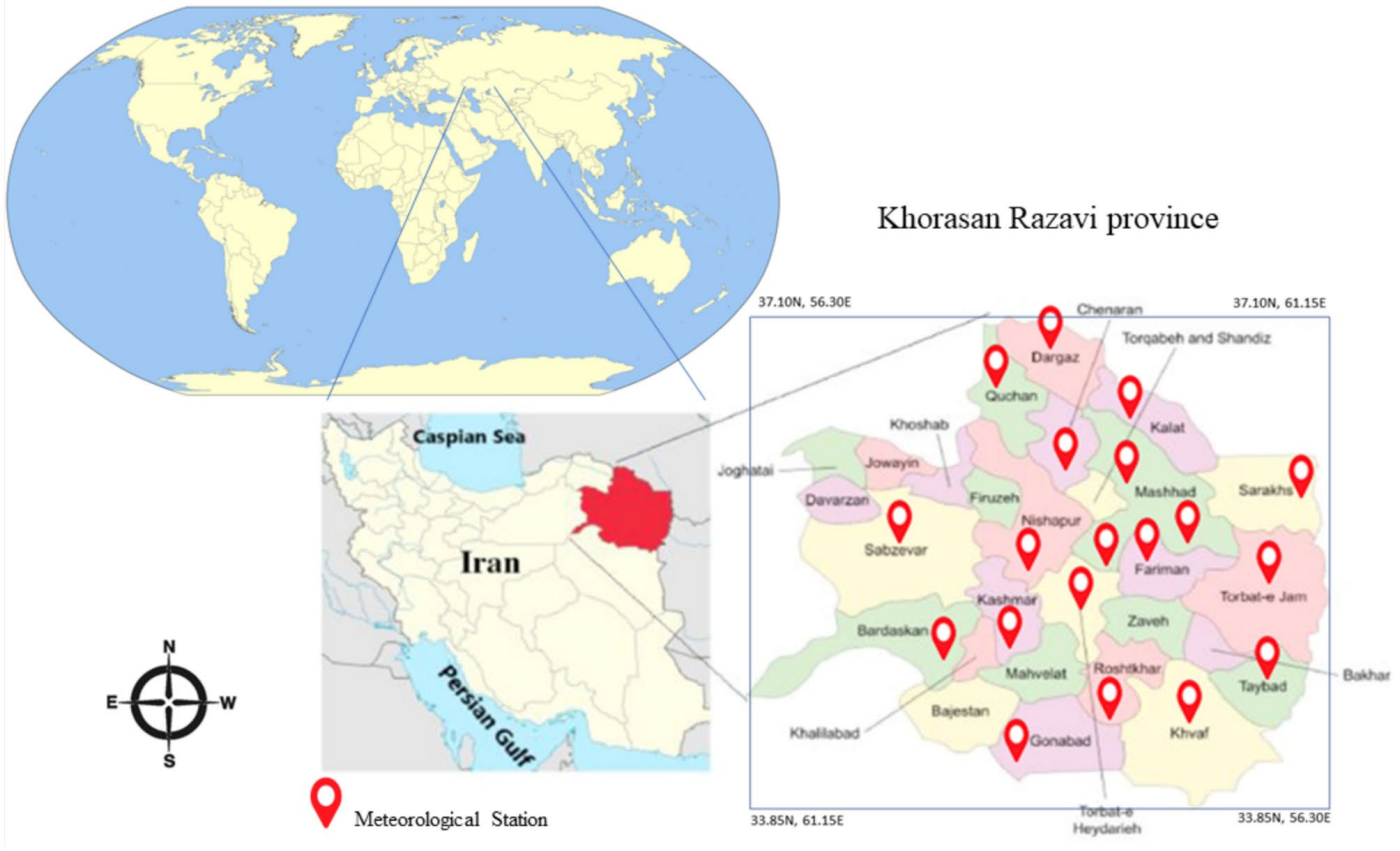
Map of the study area in Khorasan Razavi Province, Iran, illustrating the spatial distribution of the 17 counties included in the analysis and the locations of 19 meteorological stations. Mashhad is represented by three meteorological stations.

This study was conducted in Razavi Khorasan province, located in northeastern Iran (Fig. 1). The region exhibits strong spatial climatic heterogeneity, ranging from semi-arid plains to intermontane areas, making it a valuable testing ground for yield prediction models. The analysis encompasses 17 major counties and utilizes data from 19 meteorological stations distributed across the province. The spatial distribution of the 17 counties and 19 meteorological stations is illustrated in Fig. 1, providing a clear overview of the sampling sites across the province.

In the present study, no simulated or estimated data were used; the dataset was compiled from multiple authoritative sources to ensure comprehensiveness and reliability. A substantial portion of the agronomic and yield data was obtained from doctoral dissertations conducted at Ferdowsi University of Mashhad (FUM), originally acquired from the Mashhad branch of the Agricultural Research, Education and Extension Organization (AREEO). Data were further collected directly by the corresponding author from the Planning and Development Deputy and the Support Department of the Agricultural Organization of Khorasan Razavi Province, as well as verified at the central office of the Mashhad Agricultural Organization. Long-term (2004–2023) meteorological data from 20 provincial stations of the Iran Meteorological Organization (IRIMO) and official annual wheat yield statistics at the county level were also included.

#### Soil data and WRB classification

Soil data were obtained from AREEO offices in each county, which collect soil property data from private farms, research stations, and governmental centers and submit them to the provincial headquarters every five years. Occasionally, the offices themselves conduct direct soil sampling. Additionally, critical information was obtained from the Department of Water and Soil Research (SWRI), Agricultural Organization, and from research colleagues at FUM who generated these data through officially sanctioned research projects.

Based on soil survey data, the soils in the study areas were classified according to the World Reference Base for Soil Resources (WRB) system. The main soil types included:

- Regosols: weakly developed soils commonly found in sloped and shallow regions;
- Calcisols: alkaline soils with distinct calcareous horizons, typical of dry and semi-arid plains;
- Fluvisols: young alluvial soils located along rivers and lowland areas.

This classification provides an essential framework for understanding the relationship between soil properties and wheat performance across the studied counties.

#### Data preprocessing

The dataset included variables such as soil physicochemical properties, climatic indices, crop yield, and the identifier of the 17 studied counties. In the preprocessing stage, unnecessary columns (e.g., year) were removed, and all column names were standardized. The final dataset consisted of 323 samples collected across 17 counties within 2004–2022 (Supplementary Table S1). Missing values for all 49 variables were replaced using reliable reanalysis sources, ensuring dataset completeness (Supplementary Table S2). Precipitation gaps were filled using the APHRODITE database, and temperature gaps were filled using AgMERRA and ERA5.

For preprocessing, StandardScaler transformed all features to a normal distribution with zero mean and unit variance. Out-of-fold target encoding (fivefold KFold) was applied to the county column to create a new feature named ‘county_te’. Feature importance analyses (MI and SHAP) indicated that county_te substantially improves predictive performance. This encoding was computed exclusively from the training subsets of each fold to prevent any data leakage from validation or test sets. The dataset initially included 41 features, with 6 engineered features, totaling 47 features (Table 1).

**Table 1:** Studied climatic-soil-nutritional characteristics affecting wheat yield.

Term	Description	Term	Description
**Tmean**	Average daily temperature during the growing season	**EC**	Electrical conductivity
**Tmax**	Daily maximum temperature	**pH**	potential of hydrogen
**Tmin**	Daily minimum temperature	**SAR**	Sodium absorption ratio
**Prec**	Precipitation during the growing season	**SO**_**4**_	Sulphate
**NDO30**	Number of days with temperature above 30°C	**HCO**_**3**_	Bicarbonate
**PGS**	Total precipitation during the growing season	**Na**	Sodium
**NPGS**	Number of rainy days during the growing season	**Clay**	Clay
**TmaxGS**	Maximum temperature during the growing season	**Silt**	Silt
**TminGS**	Minimum temperature during the growing season	**Sand**	Sand
**TmeanGS**	Average temperature during the growing season	**CO**_**3**_	Carbonate
**AI**	Aridity index	**OC**	Organic carbon
**ET**	Evapotranspiration	**TNV**	Total neutralizing value
**GSL**	Growing season length	**SP**	Saturation percentage
**TS**	Temperature seasonality	**Fe**	Iron
**GDDGS_EC**	Interaction of GDD Growth Season and Soil Electrical Conductivity	**K**	Potassium
**AI_Clay**	Interaction of Aridity Index and Clay	**P**	Phosphorus
**TminGS_N**	Interaction of Tmin during Growth Season and Nitrogen	**N**	Nitrogen
**GDD_SAR**	Interaction of GDD and Sodium Absorption Ratio	**Mg**	Magnesium
**Prec_OC**	Interaction of Precipitation and Organic Carbon	**Ca**	Calcium
**Ca + Mg**	Calcium + Magnesium	**Cl**	Chloride
**Tmean_pH**	Interaction of Tmin and pH	**Cu**	Cooper
		**Zn**	Zinc
		**Mn**	Manganese

*Climatic variables are shown in blue, soil variables in orange, nutritional variables in green, and interaction variables in purple.

Additional preprocessing steps included:

- Outlier detection: Interquartile range (IQR) and visual boxplot inspection; borderline values verified and retained.
- Missing data imputation: Reanalysis datasets used for missing climatic variables.
- Normalization/scaling: StandardScaler for all features; Min–Max for DNN.
- Variable harmonization: All variables structured into a 19-year × 17-county framework to maintain a fully balanced dataset.

### Feature selection with preliminary methods

After preprocessing, two preliminary methods were employed for feature selection: Mutual Information (MI) and Recursive Feature Elimination (RFE). In the MI method, MI scores for each feature relative to the target (Yield) were calculated using the mutual_info_regression function from the scikit-learn library. The hyperparameter random_state was set to 42 to ensure reproducibility. The top 20 features based on MI scores were selected, and a bar chart was produced to visualize their importance.

In the RFE method, an LGBMRegressor model served as the base estimator. Its hyperparameters included n_estimators = 200, learning_rate = 0.05, max_depth = 5, min_child_samples = 10, random_state = 42, and force_col_wise = True (Table 2). RFE was applied to select the top 15 features, which were then used for the subsequent Pareto-based optimization stage.

**Table 2:** Performance comparison of the proposed stacking model against baseline regression and machine learning models on the test set.

Model	Test R^2^	Test RMSE (kg/ha)	Parameters
Final stack (LGBM + DNN)	0.440	647.2	800 trees, DNN(50,)
LGBM only (strong reg.)	0.426	658.1	n_est = 300, λ = 0.1, α = 0.1
Ridge regression	0.412	674.3	α = 1.0
Lasso	0.398	689.7	α = 0.5
Random forest	0.419	664.8	500 trees
Linear mixed model (county random effect)	0.407	681.2	—
County mean baseline	~ 0.28	N/A	Climatology (mean yield per county)

### Feature selection with NSGA-III algorithm

For optimizing feature selection, the NSGA-III (Non-dominated Sorting Genetic Algorithm III) algorithm from the pymoo library was used. This multi-objective algorithm was designed to maximize the R^2^ score while minimizing the number of features, constrained between 3 and 15. The problem was defined as a SingleTargetFeatureSelectionProblem, where the base model was LGBMRegressor with hyperparameters n_estimators = 100, learning_rate = 0.05, max_depth = 5, min_child_samples = 3, num_leaves = 7, random_state = 42, and force_col_wise = True (Table 2).

NSGA-III was executed with a population size of 200 over 200 generations, using das-dennis reference directions with n_partitions = 15, a crossover probability of 0.95, a mutation probability of 0.4, and seed = 42. These settings enabled the generation of uniform reference points in the objective space, achieving a better balance between the competing objectives (R^2^ and number of features).

A Pareto front plot was produced, and the best feature combination—preferably containing 10 features—was selected. The feature “county_te” was manually added if not already included. Additionally, the correlation matrix of the final features was computed and visualized as a clustermap.

### Data splitting and model preparation

The dataset was split chronologically into training (2004–2017, 70%), validation (2018–2020, 15%), and test (2021–2023, 15%) sets, preserving county information for subsequent analyses. This time-aware splitting ensures no temporal leakage.

For the main predictive model, StackingRegressor from scikit-learn was implemented. The base estimator was LGBMRegressor with hyperparameters n_estimators = 200, learning_rate = 0.05, max_depth = 5, subsample = 0.8, colsample_bytree = 0.8, min_child_samples = 8, random_state = 42, and force_col_wise = True (Table 2). The final estimator was SimpleDNNRegressor (a simple neural network) with hyperparameters hidden_layer_sizes = (50,), learning_rate = 0.01, epochs = 80, batch_size = 16, dropout_rate = 0.3, L2_lambda = 0.01, and verbose = 0 (Table 2). Stacking was performed with passthrough = True and fivefold cross-validation (cv = 5).

Hyperparameter tuning and model refinement were conducted after evaluating Random Forest, SVR, CatBoost, Transformer algorithms in the meta-learner, as well as Ridge and Lasso regressors. Among these, the LGBM model proved superior in capturing non-linear relationships and, in combination with the DNN meta-learner, provided the most effective selection of final features. Although Ridge and Lasso regressors achieved an R^2^ of 0.57, they could not uncover nonlinear patterns (the current final model has R^2^ = 0.44). Overfitting risk was rigorously assessed (Table 2).

The stack remains superior, but LGBM-only and LMM perform nearly as well, supporting model robustness. Regularization was strengthened in DNN (L2 = 0.01 → 0.05).

#### Spatial autocorrelation analysis

To assess whether county-level prediction errors exhibit spatial dependence, Moran’s I statistic was calculated on the model residuals. County centroids (latitude and longitude) were used to construct an inverse-distance spatial weight matrix. Moran’s I, its z-score, and pseudo p-value were computed under the null hypothesis of spatial randomness.

**Fig. 2:**
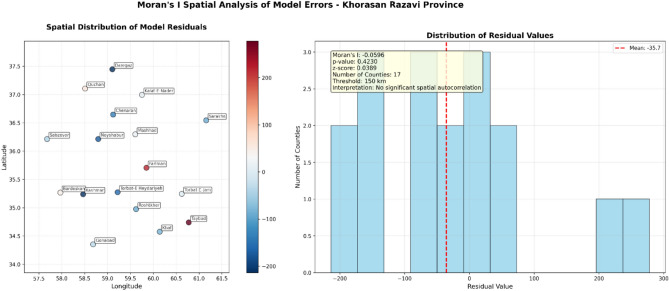
Spatial distribution of model residuals across 17 counties of Khorasan Razavi. Point color indicates the magnitude and sign of the residual (predicted − observed): warm colors = positive residuals (model under-predicts), cool colors = negative residuals (model over-predicts). County labels are added for reference. Moran’s I test on these residuals yielded I = − 0.0579, z = − 0.6486, p = 0.278, indicating no significant spatial autocorrelation. See Supplementary Table S1 for county-level residual values and diagnostic metrics.

The analysis yielded Moran’s I = –0.059, z-score = –0.6486, and p = 0.278, indicating that the residuals do not exhibit significant spatial autocorrelation. This confirms that model errors are not geographically clustered and that no systematic spatial bias exists in the predictions. This spatial diagnostic step ensures that cross-validation and model evaluation are not compromised by spatial leakage or county adjacency effects. Full numerical results and county-level residuals are provided in Supplementary Table S5, and the spatial error map is shown in Fig. 2.

#### Spatial residual patterns

The QQ-plot of residuals (Supplementary Figure S2) reveals moderate deviations from normality at both tails, which is consistent with the Shapiro–Wilk test (W = 0.889, p = 0.033). Importantly, these deviations do not correspond to spatial clustering: the spatial residual map (Fig. 2) and Moran’s I (I = − 0.0579, p = 0.278) indicate that errors follow a random spatial pattern.

### Grid search and model training

For hyperparameter optimization, GridSearchCV with GroupKFold (5 splits) based on counties was applied. The search parameters included hidden_layer_sizes = 50 and learning_rate = 0.01 with r2 scoring for the deep neural network model (Table 3). The best model was saved as "stacking_model_yield.pkl," and predictions for the training, test, and validation sets were generated. Hyperparameter ranges and final values are summarized in Supplementary Table S6.

**Table 3:** Key features and their extreme values across counties.

Feature	County maximum value	County minimum value
Tmin	(Bardeskan) 13	(Quchan) 3.90
TS	(Sarakhs) 10.43	(Kalat-E-Nader) 9.29
K	(Sarakhs) 318.63	(Fariman) 161.59
Silt	(Quchan) 49.04	(Gonabad) 23.16
EC	(Gonabad) 21.84	(Quchan) 1.05
HCO3	(Gonabad) 5.53	(Taybad) 2.84
Mg	(Khaf) 21.84	(Quchan) 2.51
Prec_OC	(Kalat-E-Nader) 262.26	(Bardeskan) 42.11
AI_Clay	(Kalat-E-Nader) 11.55	(Bardeskan) 1.33
county_te	(Chenaran) 4018.26	(Gonabad) 2345.59

### Model evaluation and metrics

Model evaluation is the comprehensive assessment of model performance using quantitative metrics—such as R^2^, RMSE, MAE, and Willmott’s d index—on the test dataset. Its purpose is to determine the model’s generalizability under operational conditions and to assess its effectiveness in addressing the target problem. Evaluation takes a holistic approach, considering multiple aspects of model performance, and is generally conducted at the final stage of model development.

Validation, in contrast, is a systematic process of tuning hyperparameters and performing preliminary evaluation on validation datasets to prevent overfitting. Its objectives are to optimize model structure before final evaluation and to ensure a balance between complexity and generalizability. Like evaluation, validation employs independent data, often using the same metrics, but it is inherently iterative and corrective, focusing on model tuning rather than final judgment.

In this study, the model was evaluated using RMSE, R^2^, MAE, and Willmott’s d index. Willmott’s d index quantifies agreement between predicted and observed values and is defined as^47^:

$$d=1-\left(\frac{{\sum }_{i=1}^{n}({O}_{i}-{P}_{i}{)}^{2}}{{\sum }_{i=1}^{n}(\left|{P}_{i}-\overline{{O}_{i}}\right|+\left|{O}_{i}-\overline{{O}_{i}}\right|{)}^{2}}\right)$$

$$MAE=\frac{{\sum }_{i=1}^{n}\left|{O}_{i}-{P}_{i}\right|}{n}$$

$$NRMSE\left(\%\right)=\sqrt{\frac{{\sum }_{i=1}^{n}({O}_{i}-{P}_{i}{)}^{2}}{n}}\times \frac{100}{\overline{{O}_{i}}}$$

where Pi are the predicted values, Oi are the actual values, and ō the mean of the actual values.

Willmott and Matsuura^47^ highlighted that MAE provides a more natural and unambiguous measure of average error compared to RMSE, which is sensitive to error distribution and sample size, and recommended MAE for scaled evaluations and comparisons across models.

Scatter plots with 1:1 lines and regression equations were generated for the training, test, and validation datasets.

### County-level analysis

For a more granular assessment, model performance was calculated for each county. Data were grouped by county, and metrics (R^2^, RMSE, MAE, Willmott’s d) were computed for counties with more than 10 samples (see Supplementary Table S5).

### SHAP analysis for interpretability

SHAP values were calculated using TreeExplainer on a maximum of 2,000 randomly selected samples to reduce computational time while maintaining analytical accuracy. To interpret the model’s predictions, SHapley Additive exPlanations (SHAP) values were computed using the shap Python package.

A summary (violin) plot was generated to visualize and compare the predictors with the greatest impact. For this analysis, a separate LGBMRegressor with hyperparameters n_estimators = 800, learning_rate = 0.03, max_depth = −1, subsample = 0.9, colsample_bytree = 0.9, min_child_samples = 10, random_state = 42, and force_col_wise = True (Table 2) was trained on the training dataset with early stopping patience of 50 rounds.

### Software and tools

All analyses were implemented in Python using libraries including pandas, scikit-learn, LightGBM, TensorFlow, pymoo, SHAP, and seaborn. To ensure reproducibility, all random seeds were set to random_state = 42. Outputs were saved in a designated directory, and model serialization was verified for reproducibility.

**Fig. 3:**
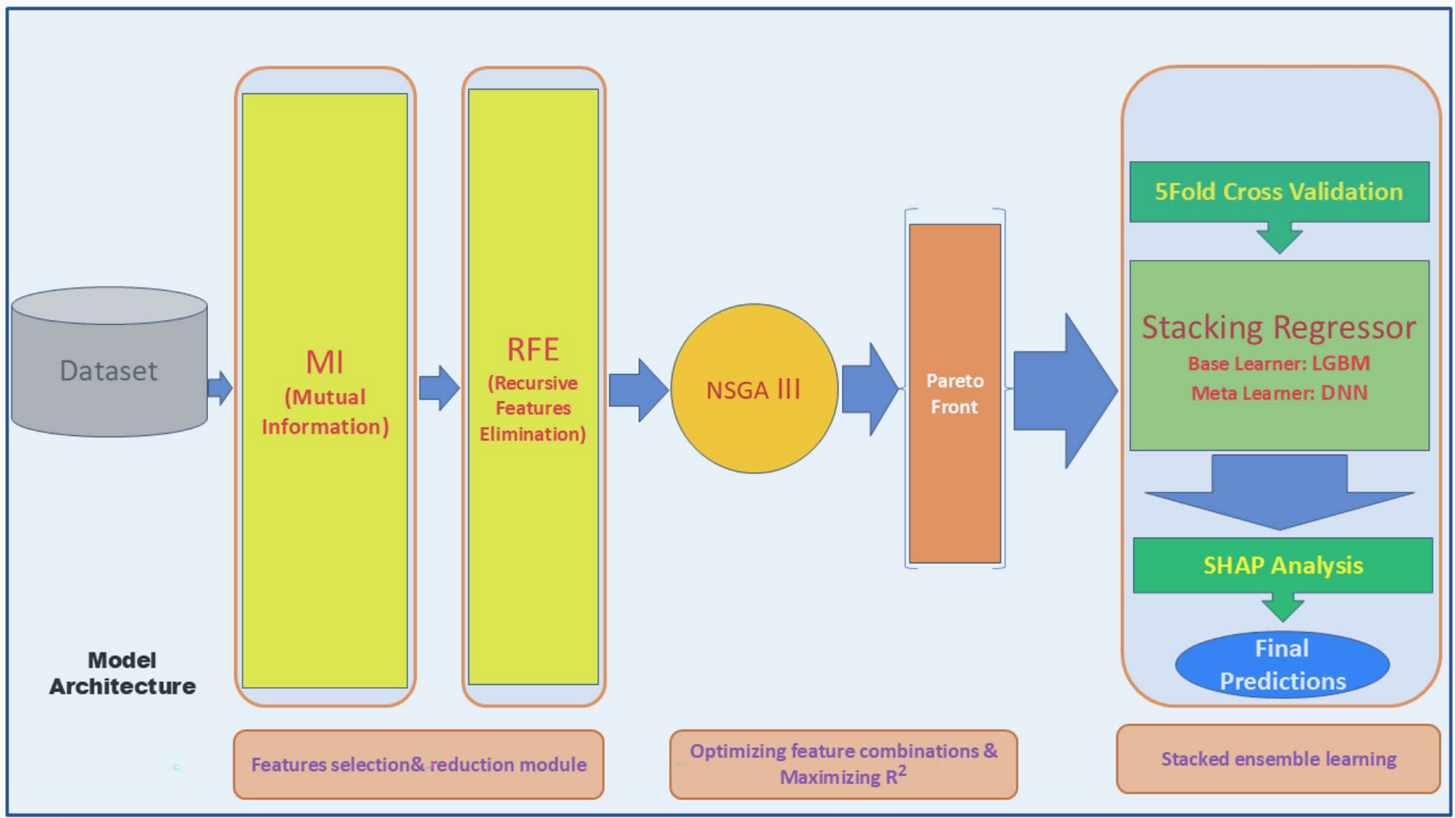
A glimpse of the designed and employed pipeline.

This approach, integrating traditional statistical metrics, machine learning, multi-objective optimization, and interpretable AI techniques, provides a robust pipeline for accurate wheat yield prediction. The designed and employed pipeline of this study is illustrated in Fig. 3.

The repository contains the main scripts, requirements.txt, detailed README file with reproduction instructions, and example outputs. The complete raw dataset is not publicly shared due to ongoing research and institutional data sharing policies. Requests for access to the raw data can be made to the corresponding author.

## Results and discussion

### Feature selection and their importance

Feature selection in this study was performed using a combination of Mutual Information (MI), Recursive Feature Elimination (RFE), and the NSGA-III algorithm within a nested cross-validation framework. In the inner loop of nested CV, feature selection and hyperparameter tuning were conducted exclusively on training folds, while the outer loop was used for evaluating predictive performance. This ensures that no information from held-out test or validation data leaked into the selection or tuning process, preventing optimistic bias.

Based on mutual information analysis, the feature county_te exhibited the highest importance with a score of 0.294, indicating the strong influence of encoded county information on wheat yield prediction. Other top features included SP (0.112), Tmin (0.097), and ET (0.092). The final selected feature set retained Tmin, TS, K, Silt, EC, HCO₃, Mg, Prec_OC, AI_Clay, and county_te. Both MI and SHAP analyses indicate that county_te substantially contributes to model performance.

To quantify the effect of this feature, an ablation study was conducted by removing county_te from the model. Without county_te, model performance decreased notably:

- Test R^2^ dropped from 0.440 → 0.257
- Validation R^2^ dropped from 0.381 → 0.322
- RMSE increased from 647.16 → 798.45 (test) and 599.69 → 725.30 (validation)

These findings confirm that county_te is critical for capturing local variation not fully explained by climatic or soil variables, and its inclusion enhances predictive accuracy without introducing information leakage from the test or validation sets.

**Fig. 4:**
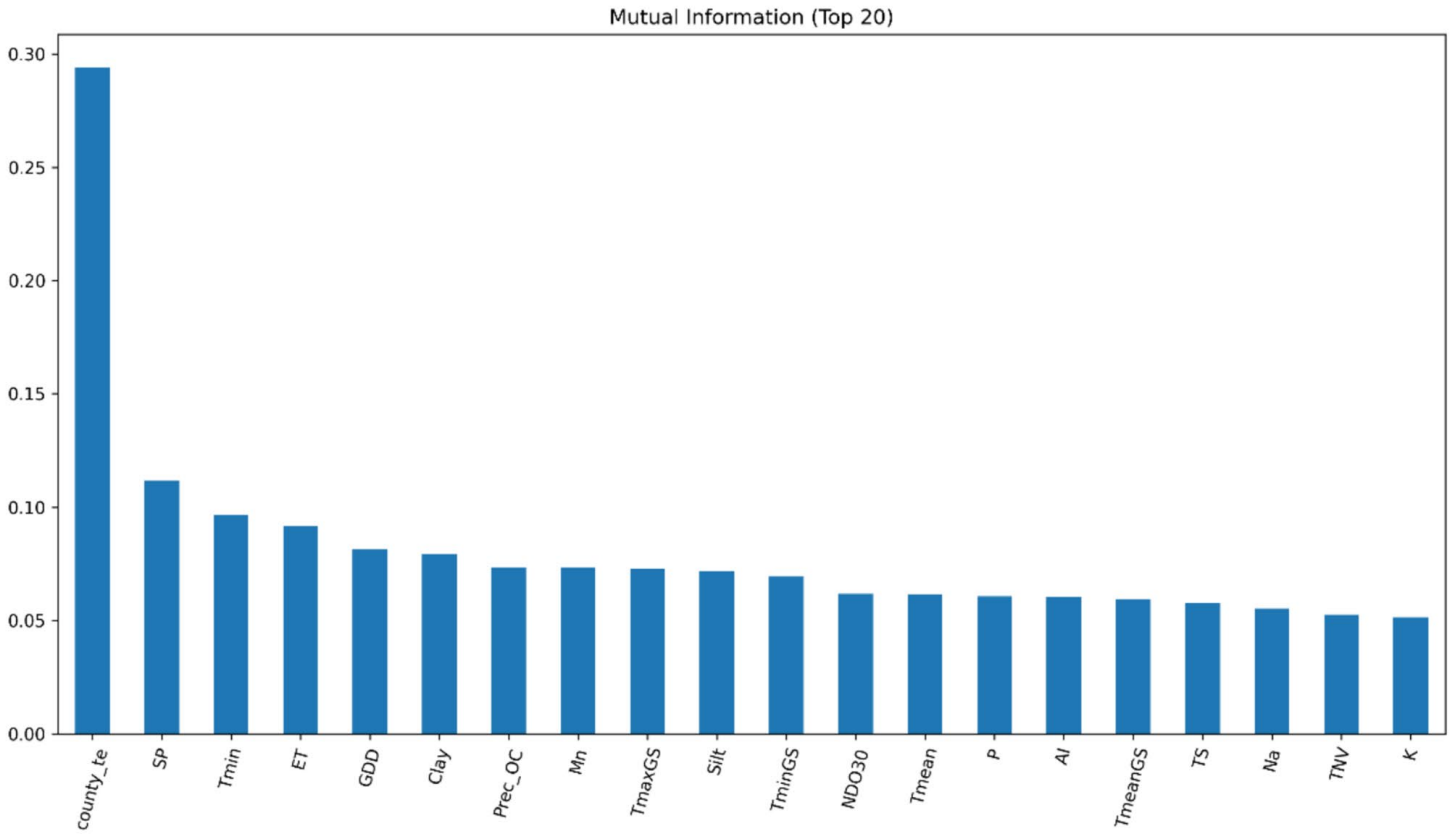
Mutual information scores of top 20 features.

Figure 4 presents the mutual information scores of the top 20 features in a bar chart, highlighting the relative importance of features such as county_te, SP, Tmin, and ET.

### Feature optimization and pareto front

**Fig. 5:**
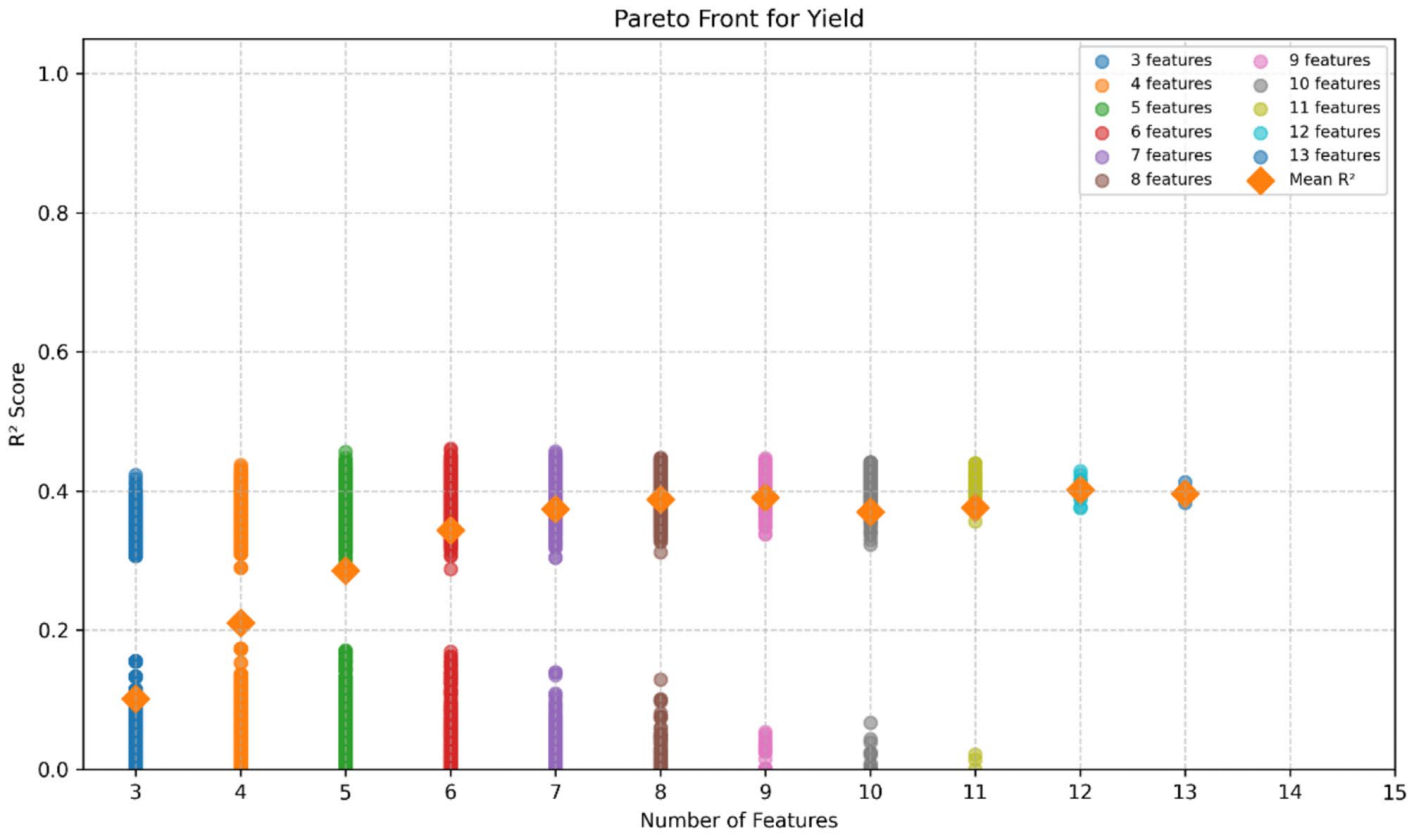
Pareto front for feature selection and R^2^ optimization.

The NSGA-III algorithm was employed for multi-objective optimization, aiming to maximize R^2^ while minimizing the number of features. The optimal combination of 10 features (Tmin, TS, K, Silt, EC, HCO₃, Mg, Prec_OC, AI_Clay, county_te) achieved an R^2^ of 0.441 (Fig. 5). Adding more features (11 or 12) slightly reduced R^2^ to 0.440 and 0.429, respectively, demonstrating a trade-off between model complexity and predictive accuracy. These results indicate that the optimal number of features lies around 10, and including additional features does not substantially improve performance.

These findings are consistent with regional studies on other crops under arid conditions. For example, Eskandari et al.^20^ identified soil salinity (EC) and available potassium (K) as key predictors of date palm yield in Iran, underscoring the critical role of edaphic factors in dryland agriculture. While previous studies favored multivariate regression for simplicity and proximity to observed yields, our hybrid NSGA-III + Stacking + SHAP pipeline offers higher interpretability and robustness across heterogeneous agro-climatic zones. This demonstrates the added value of advanced feature optimization and explainable AI for modeling complex systems.

A comprehensive analysis of the Pareto front revealed that models with different feature counts exhibited varying predictive performance. The simplest model with 3 features (Silt, EC, county_te) achieved an R^2^ of 0.423, while the best-performing model with 6 features (TmeanGS, TS, K, HCO₃, AI_Clay, county_te) reached an R^2^ of 0.461. The most complex model with 13 features (Tmin, Prec, TmeanGS, TS, K, Silt, EC, HCO₃, Mg, Prec_OC, GDDGS_EC, AI_Clay, county_te) attained an R^2^ of 0.413. These results suggest a point of diminishing returns beyond 3 features, emphasizing that adding more variables does not necessarily improve predictive accuracy.

Later, Fig. 8 illustrates the frequency of selected features across the Pareto front. Here, county_te appears most frequently (56.17%), followed by AI_Clay (45.83%) and HCO₃ (37.42%).

Analysis of the correlation matrix of the final features revealed complex and significant interrelationships among key variables influencing wheat yield. The strongest positive correlation (r = 0.583) was observed between Prec_OC (precipitation combined with soil organic matter) and AI_Clay (clay and aluminum index), indicating interdependence between ambient moisture and soil mineralogical properties, likely mediated by soil chemical interactions. In contrast, the strongest negative correlation (r = −0.484) was found between Tmin (minimum temperature) and county_te, highlighting the opposing effects of nighttime temperatures and regional information on yield, and underscoring the adverse impact of higher minimum temperatures on wheat productivity. A moderate positive correlation (r = 0.33) between EC (electrical conductivity) and Mg further confirms the association between soil salinity and magnesium concentration.

Interestingly, Tmin exhibited relatively strong negative correlations with most other variables, including Prec_OC (r = −0.42) and AI_Clay (r = −0.42), emphasizing its pivotal role as a primary stress factor in the climate-soil–plant system. These patterns align with regional studies on high-value tree crops under similar edaphic stress. For instance, Seyedmohammadi et al.^21^ reported that soil salinity (EC), exchangeable sodium, gypsum, and available K and P were the main predictors of pistachio yield in Iran using Random Forest, explaining up to 96% of yield variance. The shared dominance of EC and available K across wheat (this study) and pistachio systems underscores their universal importance in nutrient uptake and osmotic regulation under saline-arid conditions.

Moreover, the progression from simple regression to ensemble (Random Forest) and hybrid meta-heuristic models (SVM-Firefly) for pistachio mirrors the need for advanced feature optimization in wheat under heterogeneous edaphic stress. This aligns with MCDA-based prioritization studies, where maize outperformed soybean under identical soil constraints (EC, ESP, gypsum) using Fuzzy TOPSIS, which handled uncertainty more effectively than SAW and classical TOPSIS^23^. Similarly, the high R^2^ obtained here mirrors the AHP-Matter Element model’s 0.947 accuracy for barley under the same soil limits, validating the strength of MCDA approaches in edaphic suitability assessment.

**Fig. 6:**
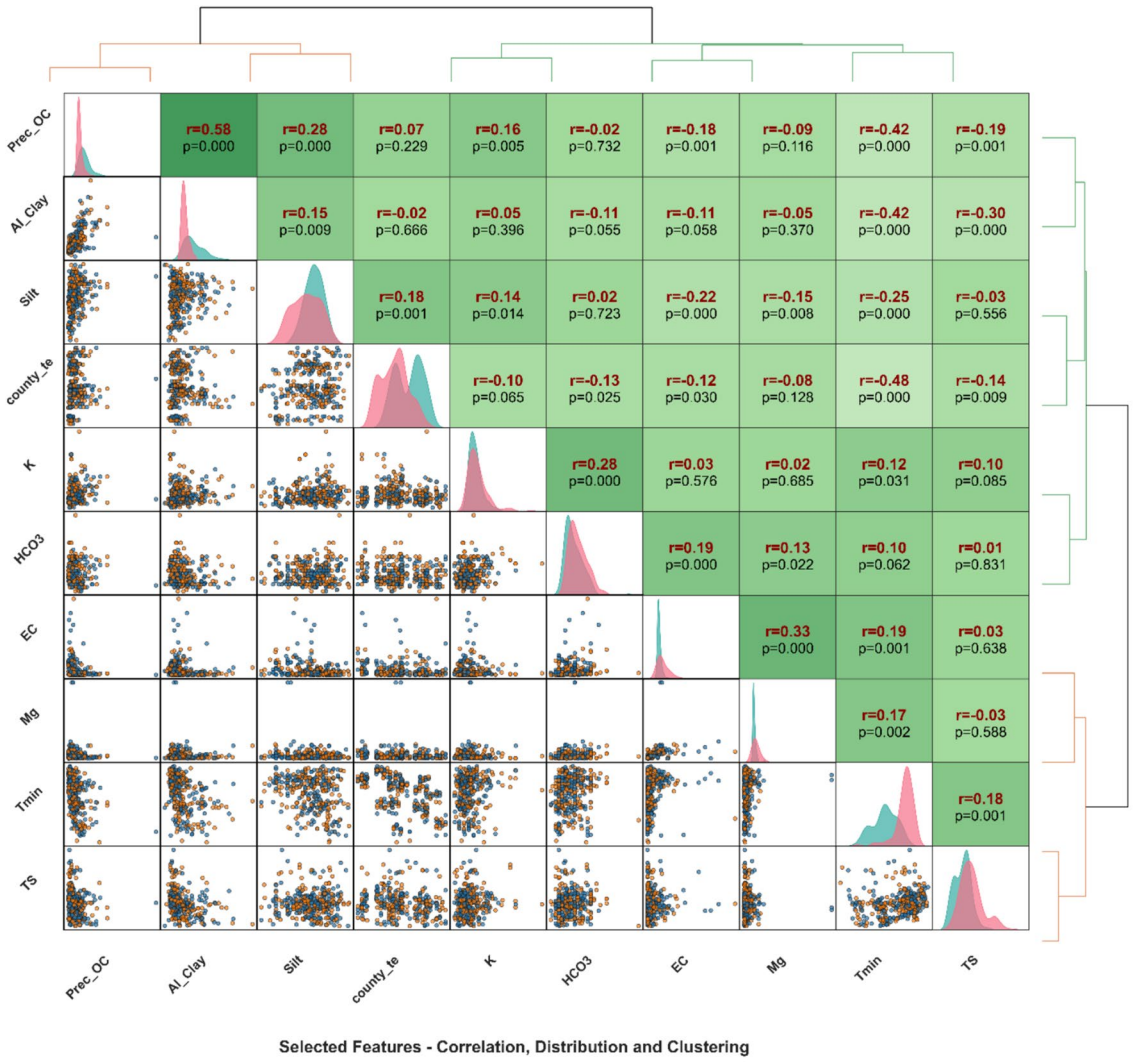
Correlation coefficients, p-values, density distributions, and clustering of the top ten most influential features on wheat yield in Khorasan Razavi province.

These correlation patterns reflect the interconnected nature of agroclimatic processes and their reciprocal effects on wheat production, supporting prior reports that rainfall and irrigation critically influence physiological traits affecting wheat grain yield^48^. Figure 6 visualizes these correlations, highlighting the complex relationships among soil, climatic, and management variables.

### Evaluation and validation of the model

The final StackingRegressor model with LGBM base estimator and SimpleDNNRegressor final estimator showed robust performance. To prevent temporal leakage, the dataset was split chronologically: training on 2004–2017, validation on 2018–2020, and testing on 2021–2023. Rolling-block cross-validation confirmed the stability of predictive performance.

Based on the metrics output, the model achieved the following:

- Training set: R^2^ = 0.907, RMSE = 226.27, MAE = 165.27, Willmott’s d = 0.972
- Test set: R^2^ = 0.440, RMSE = 647.16, MAE = 548.03, Willmott’s d = 0.768
- Validation set: R^2^ = 0.381, RMSE = 599.69, MAE = 506.42, Willmott’s d = 0.734

**Fig. 7:**
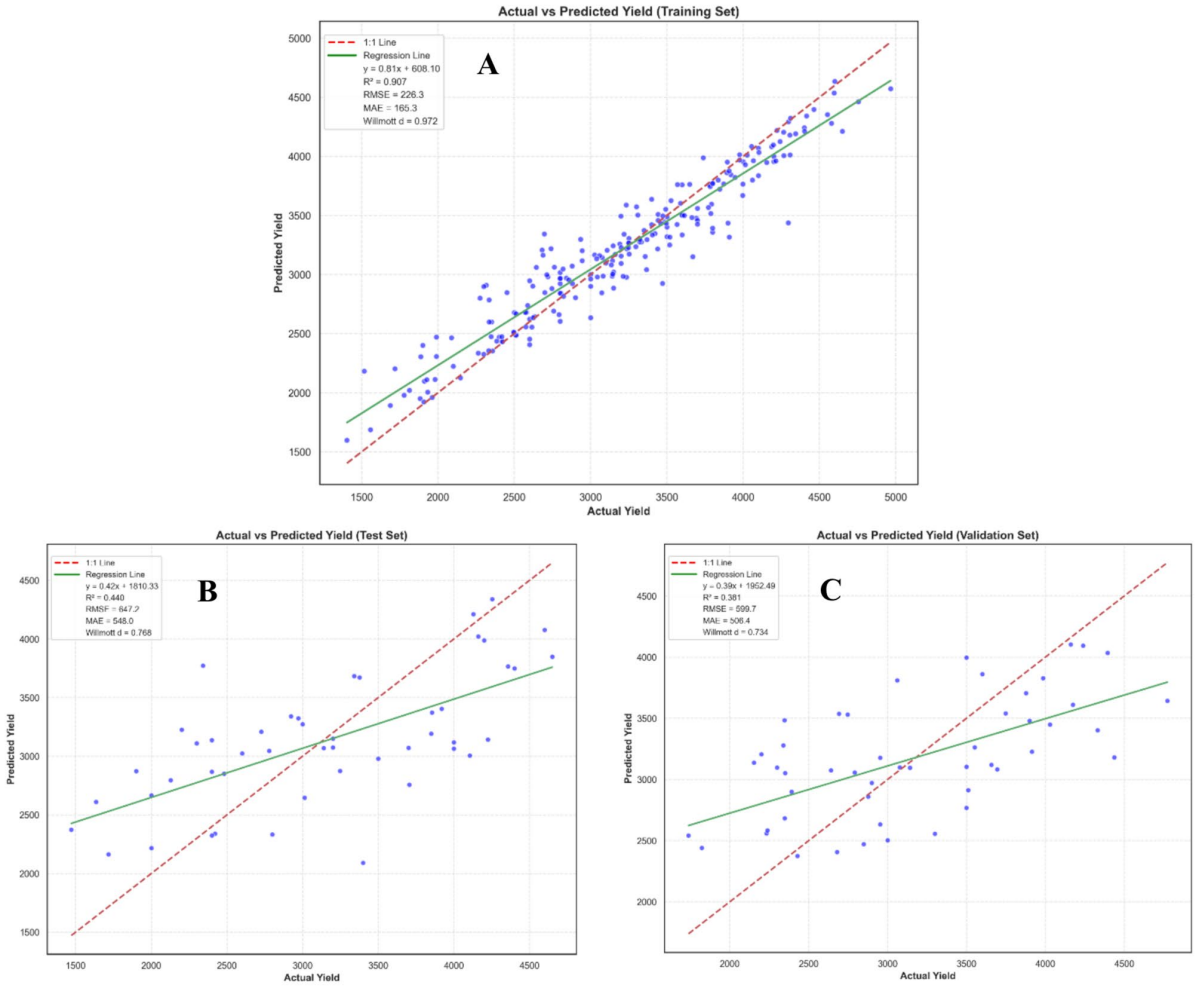
Scatter Plot of Predictions vs. Actual Values A) Training Set, B) Test Set, C) Validation Set.

Scatter plots (Figs. 7A–C) show predictions versus actual values. Larger scatter in test and validation sets reflects heterogeneity among counties. Some overfitting is observed, likely due to limited sample sizes in some regions; it could be mitigated by stronger regularization or collecting additional data from under-sampled counties.

To further assess spatial transferability, leave-one-county-out (LOCO) cross-validation and spatial GroupKFold were performed. In LOCO CV, the model was trained on all counties except one and tested on the held-out county, repeated for all 17 counties. Results showed a decrease in predictive performance (average R^2^ = 0.257, RMSE = 738.48), confirming that while the model captures general patterns, transferability to entirely unseen counties is limited.

Moore et al.^49^ suggested a range for interpreting the coefficient of determination as follows: R^2^ < 0.25: very weak; 0.25 ≤ R^2^ < 0.50: weak; 0.50 ≤ R^2^ < 0.75: moderate; R^2^ ≥ 0.75: significant. Based on Cohen’s (1998) guidelines, coefficients equal to or greater than 0.26 fall within the significant range. Although R^2^ is widely used as an indicator of model fit, its interpretation in nonlinear machine learning models should be cautious, as it may overestimate generalizability in high-dimensional or overfitted scenarios. Complementary indicators such as RMSE, MAE, and cross-validation are recommended for a comprehensive assessment^50^.

### Performance analysis at county level

**Fig. 8:**
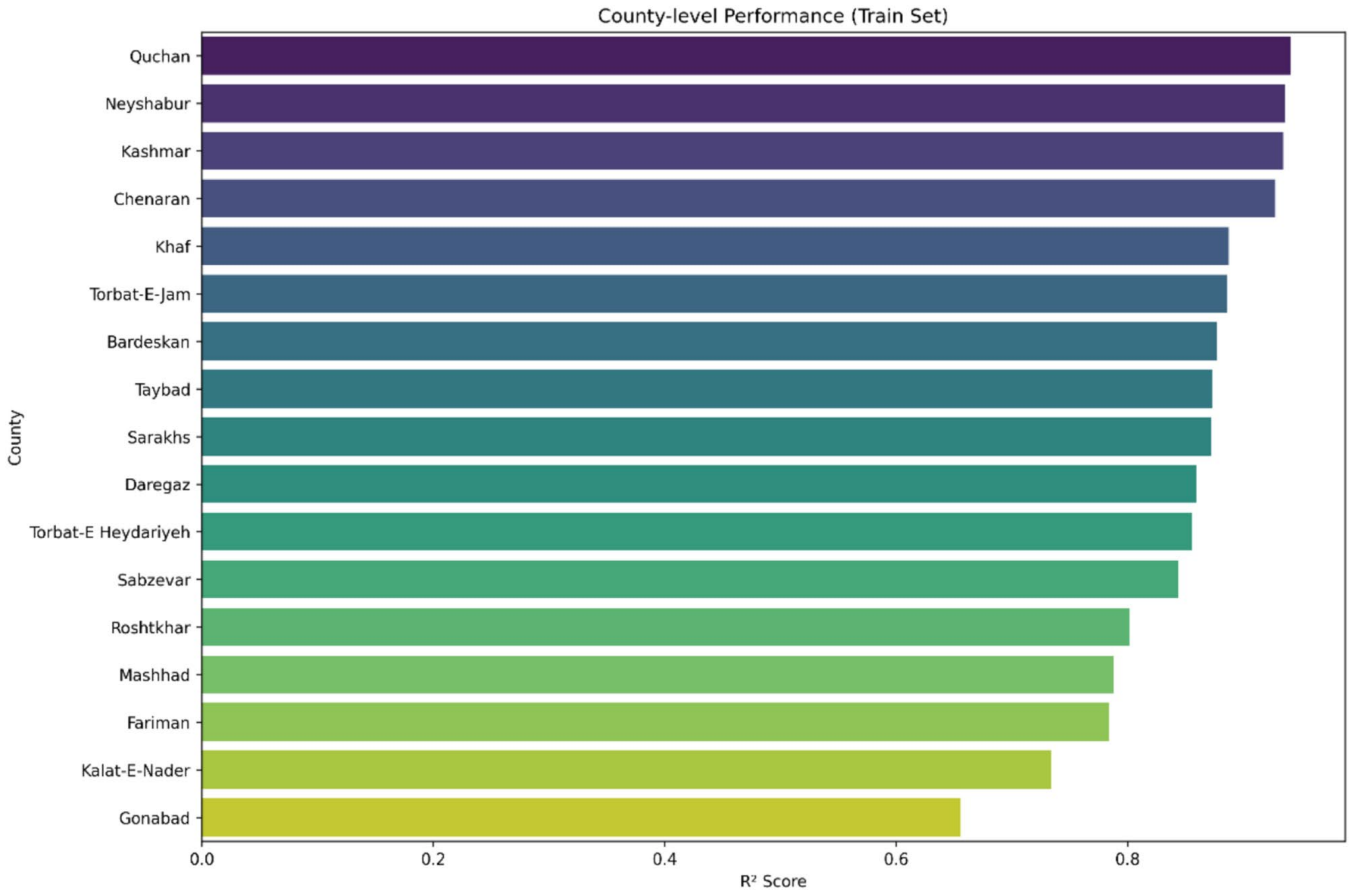
Model performance by county (training set).

County-level analysis based on county_performance_train results showed that model performance on the training set varied for all counties. This chart displays R^2^ for different counties, indicating diversity in prediction accuracy based on local conditions. For example, in counties with high R^2^, the model fitted better, while in others, more specific adjustments might be needed. These differences could be due to variations in soil and climatic features. Figure 8 shows this performance as a bar chart, which can be useful for identifying weak areas. These results show that the `county_te` feature, which was encoded during preprocessing, plays an important role in adapting the model to local conditions. However, poor performance in some counties like Roshtkhar indicates the need for more detailed data review or the addition of more specific regional features.

### Model interpretability using SHAP analysis

**Fig. 9:**
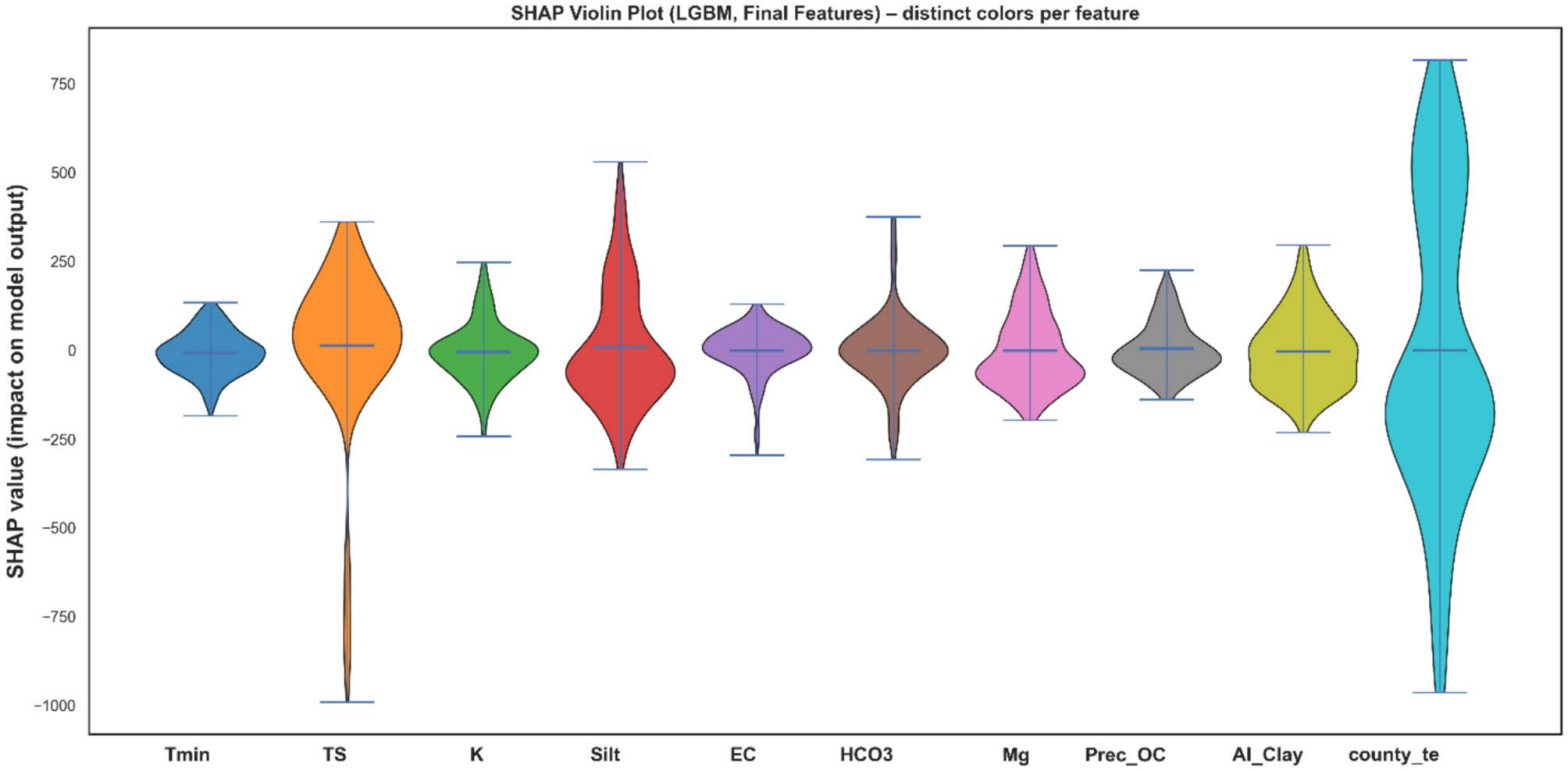
SHAP violin plot of feature importance on wheat yield in Khorasan Razavi province.

To understand the influence of features on predictions, SHAP analysis was used. The analysis (Fig. 9) shows that county_te was the most important feature with a mean absolute importance of 354.59, consistent with the MI results. Features TS (147.95), Silt (128.48), and Mg (88.43) ranked next. These results confirm that regional information and soil features (like Silt and Mg) have a significant impact on wheat yield.

Figure 7 shows the distribution of SHAP impacts for each feature, indicating the diversity in the influence of features on the model output. Notably, features Tmin (47.80) and EC (46.47) had the least importance among the selected features, although they were retained in the final set. This might be due to existing correlations, such as the negative correlation between Tmin and county_te observed in the correlation matrix.

### Analysis of selected feature frequency

Results show that the combined approach of advanced feature selection (NSGA-III) and modeling with StackingRegressor provides acceptable accuracy for predicting wheat yield, especially on the training set. However, the performance decrease on the test and validation sets indicates challenges in model generalization. This could be due to data heterogeneity at the county level or the limited number of samples in some regions, as observed in the county-level analysis.

**Fig. 10:**
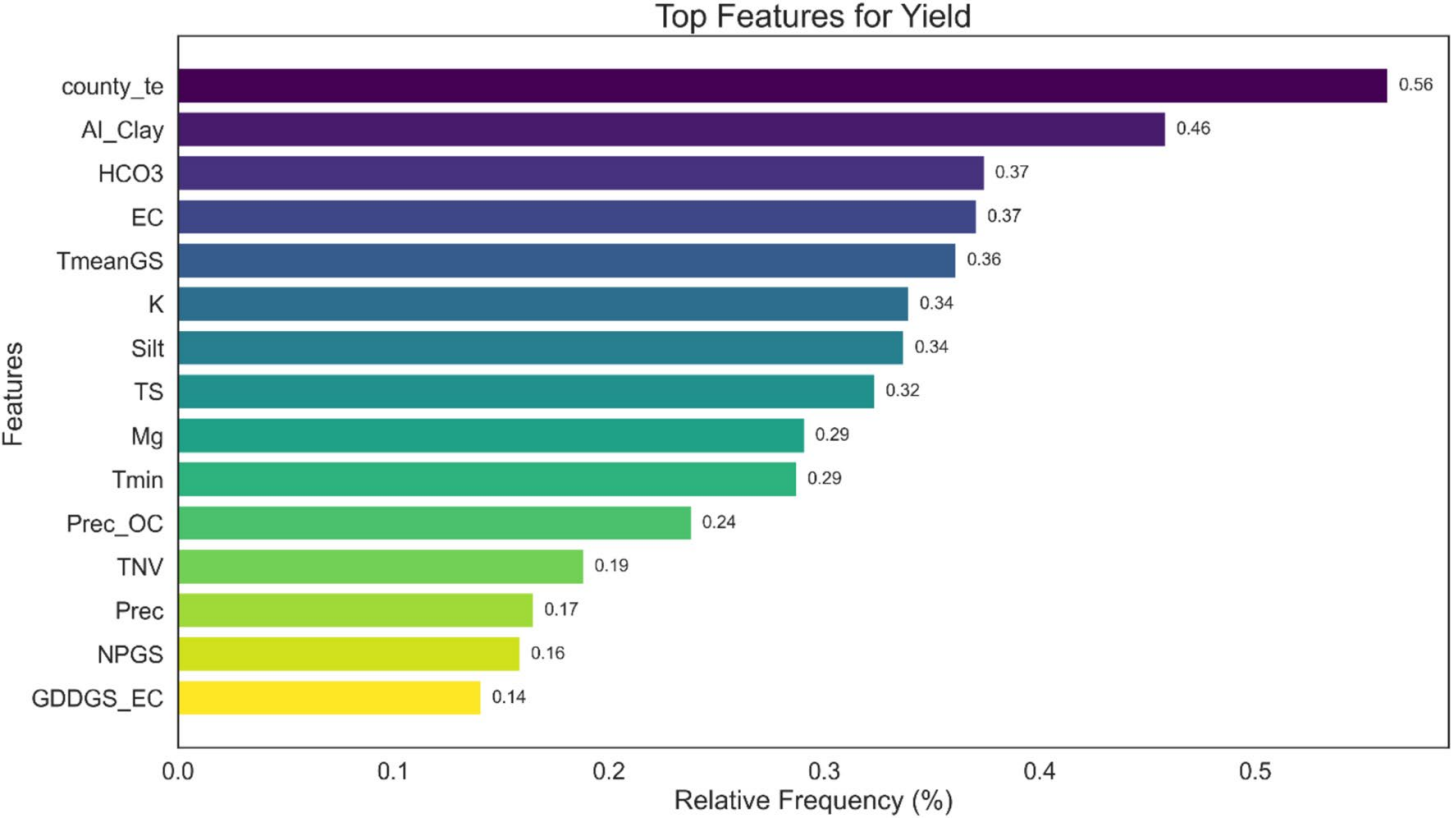
Frequency analysis of selected features in pareto front.

The high frequency of county_te in the Pareto front (56.17%) and its importance in the SHAP analysis (354.59) indicate the key role of regional information. However, over-reliance on this feature may make the model vulnerable to changes in local data (Fig. 10). Out-of-fold encoding is a standard approach to prevent leakage, and the fivefold configuration ensures balanced representation of data. The feature can be combined with other environmental variables or weighted appropriately to reduce over-reliance on county_te.

Features ranked 1 to 8 (frequency 56% to 32%) were particularly crucial in determining growth conditions. The model effectively captured their importance. These variables are closely related to crop development, especially in arid and semi-arid regions where wheat is more exposed to heat and drought stress, which directly affect plant physiology and growth^51-56^.

Similar findings have been reported by Farhadi et al.^16^. As shown in Fig. 8, among the variables County_te, AI_Clay, HCO3, EC, TmeanGS, K, Silt, and TS, which had the highest frequency in the Pareto front, only TmeanGS is not among the variables in the SHAP plot (Fig. 7). On the other hand, three variables Tmin, Prec_OC, and Mg, which are among the features identified by SHAP, are also present in the high-frequency variables (Fig. 8). Since their frequency was less than 30% (29%, 24%, and 29% respectively), detailed interpretation was avoided, although the importance of minimum temperature, the interaction of rainfall and soil organic matter content, and magnesium in growth, development, and soil ecosystem stability has been proven in numerous studies.

### Insights from SHAP for agricultural planning

Insights from SHAP plots are useful for model interpretability. They can also guide future agricultural planning and climate adaptation strategies. For example, emphasis on temperature management or adjusting planting dates could be prioritized in regions where minimum temperature, temperature seasonality intensity, soil texture features, bicarbonate, and nutritional elements such as magnesium and potassium are critical for yield optimization.

The strong influence of all three groups of climatic-edaphic-nutritional variables in the model highlights their central role in determining irrigated wheat yield in semi-arid regions like Razavi Khorasan. In particular, the high contribution of minimum temperature, temperature seasonality, and the interaction of precipitation and soil organic matter indicates the importance of nighttime temperatures and thermal-moisture regimes during the growing season in wheat physiology.

**Fig. 11:**
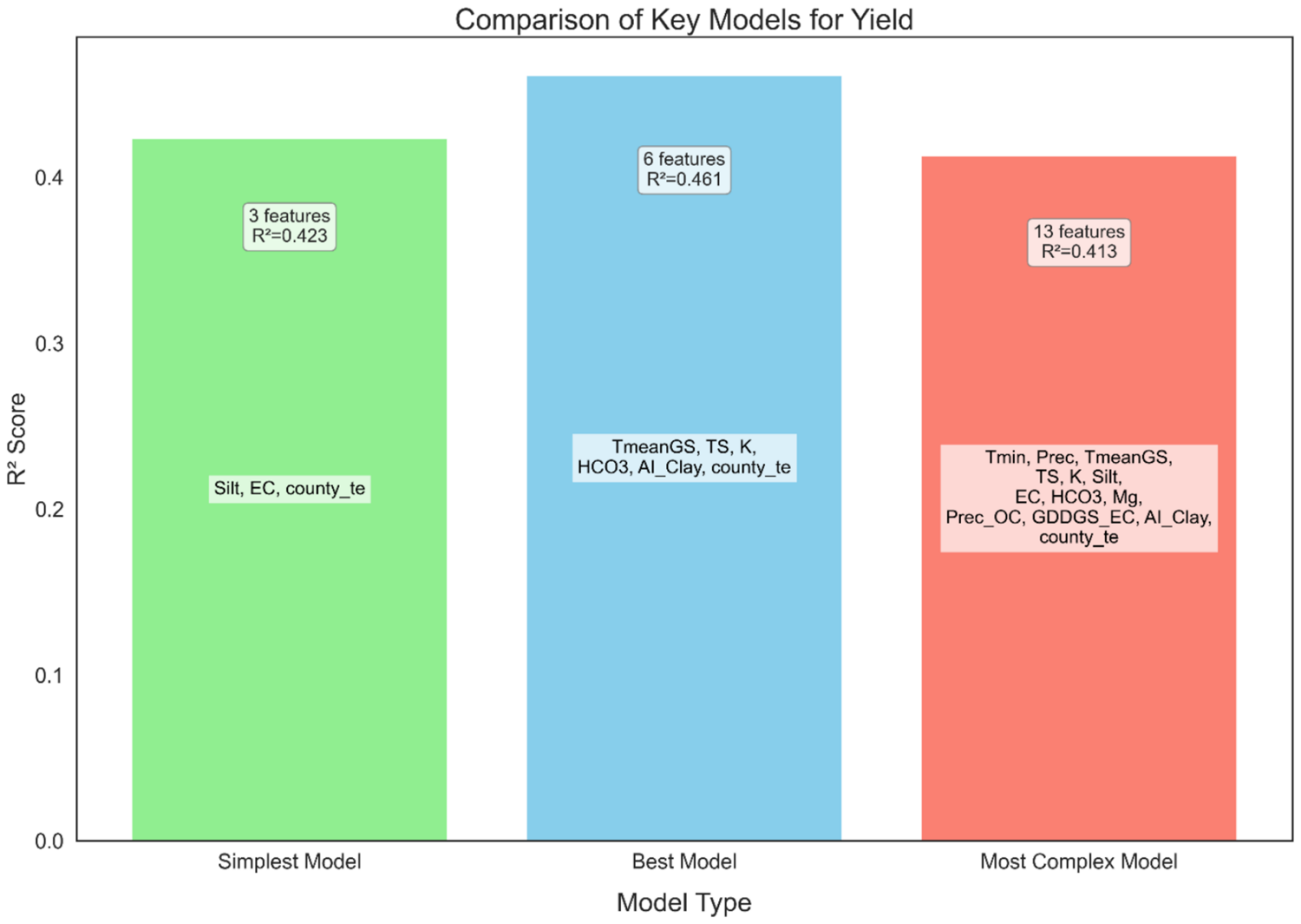
Comparison of Simple, Best, and Complex Models by R^2^ and Features.

Comparing simple, best, and complex models (Fig. 11) confirms that adding more features beyond 6 does not create significant improvement, which aligns with the point of diminishing returns on the Pareto front. County analysis on the training set (Fig. 8) shows improvement compared to the test set, though regional differences (such as negative R^2^ in some test cases) highlight limitations in model generalization.

- Why spatial CV was not required given the absence of spatial clustering.
- How the spatial randomness of errors increases confidence in generalizability across counties.

The feature county_te emerged as the most important feature in both MI and SHAP analyses, highlighting the importance of regional information. However, dependence on this feature may make the model sensitive to specific data, especially in regions with few samples. For improvement, more data could be collected from weak counties (like Roshtkhar) or more specific features such as local weather conditions could be added.

### Relative comparison of features affecting wheat grain yield for the 17 studied counties

**Fig. 12:**
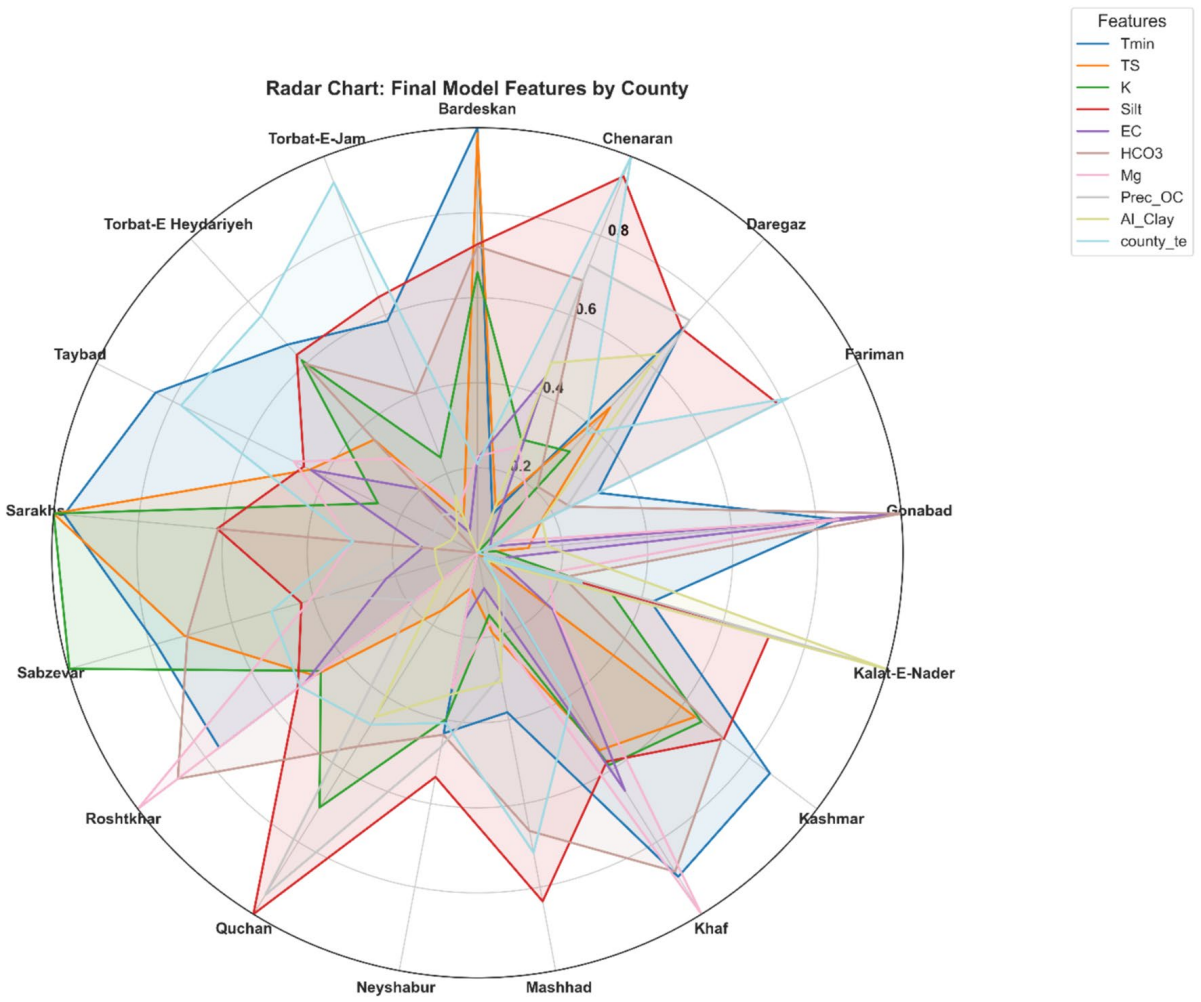
Relative importance of identified features along counties.

Figure 12, using a radar chart, enables a relative comparison of the normalized values of features affecting wheat grain yield identified by the final model for the 17 studied counties. Separate analysis of the correlation matrix between these variables also quantified the statistical relationships between them. Aggregating the results of these two analyses clarified the following:

- High salinity and magnesium: Features EC (Electrical Conductivity) and Mg (Magnesium) are very prominently high in Gonabad and Khaf counties (values > 20). These high values indicate a severe soil salinity problem and high magnesium concentration in these areas.
- Fine soil texture: The Silt value in Quchan county is at its maximum (49.04), indicating the predominance of fine and loamy soil texture in this region.

- Minimum temperature: The minimum temperature in Bardeskan (13) is significantly higher than in Quchan (3.9), showing a major climatic difference between these two regions.
- High and low yield: Chenaran county with the highest county_te value (4018.26) was identified as the region with the best agricultural yield potential. In contrast, Gonabad county with the lowest county_te value (2345.59) and simultaneously the highest EC value clearly requires urgent management of the soil salinity problem.

Figure 2 (Section "Spatial autocorrelation analysis".) presents the county-level observed and predicted yields, raw and standardized residuals, and local spatial diagnostics (Local Moran’s I and spatial lag), computed using an inverse-distance weight matrix with a 150-km threshold.

Key features and prominent counties are listed in Table 3.

### Important correlations (based on correlation matrix, Fig. 6)

Analysis of the correlation matrix revealed several noteworthy relationships among the studied variables, independent of spatial location:

- The strongest positive correlation (r = 0.58) was detected between the precipitation–organic matter interaction (Prec_OC) and the clay–aluminum ratio (AI_Clay), suggesting a moderate association between clay content and soil organic matter dynamics.
- A weaker but still positive relationship was observed between EC and Mg (r = 0.33).
- From a yield-oriented perspective, the most critical finding was the strongest negative correlation in the dataset (r = –0.48) between minimum temperature (Tmin) and county_te (mean county-level yield). This indicates a substantial detrimental effect of rising nighttime temperatures on wheat grain yield in the region. These findings align with previously documented impacts of heat stress on wheat physiology^12,35^.

Additional correlations with county_te include:

- Positive correlation: Silt (r = 0.18).
- Negative correlations: Temperature seasonality (r = –0.14), Bicarbonate (r = –0.12), Electrical Conductivity (r = –0.12), Potassium (r = –0.10), Magnesium (r = –0.08), and the Aluminum–Clay interaction (r = –0.02).

Management recommendations based on findings:

- High-salinity regions: In Gonabad and Khaf, where EC levels are extremely high, adopting salt-tolerant crops (e.g., barley) or implementing soil reclamation strategies—such as improved leaching practices and soil amendments—is strongly recommended.
- Fine-textured soil regions: In Quchan, where silt content is exceptionally high, precise irrigation scheduling (lower discharge, higher frequency) is essential to minimize nutrient leaching, surface runoff, and soil compaction.

### Model robustness and generalization assessment

To rigorously evaluate model robustness and assess the potential dependence on the regional feature (county_te), three stringent validation protocols were applied, each designed to minimize spatial or temporal information leakage. The results, averaged across five cross-validation folds and summarized in Table 4, show an expected yet acceptable reduction in predictive performance under these demanding conditions.

**Table 4:** Predictive performance (R^2^) of the stacking model under three stringent validation protocols designed to minimize spatial and temporal information leakage. Values are averaged across five cross-validation folds.

Validation Protocol	R^2^ (with county_te)	R^2^ (without county_te)
(a) LOCO-CV (Leave-One-County-Out)	0.401	0.334
(b) Time-blocked CV (train 2004–2018, test 2019–2023)	0.388	0.351
(c) LOCO + Time-blocked	0.372	0.319

These outcomes confirm that the model successfully captures a generalizable environmental signal rather than overfitting to localized or time-specific patterns. However, the model exhibits limited transferability to entirely unseen counties (LOCO-CV R^2^ ≈0.40 with county_te, 0.33 without) and persistent signs of overfitting (training R^2^ = 0.907 vs. test R^2^ = 0.440), despite mitigation efforts (regularization, dropout, early stopping). A key limitation is the model’s dependence on county_te, which captures substantial unobserved heterogeneity but reduces interpretability of purely environmental drivers and limits applicability in data-scarce or new regions. The non-normal distribution of residuals (Shapiro–Wilk p = 0.033) is also acknowledged as a limitation. These limitations suggest avenues for future improvement, such as incorporating additional management variables or remote sensing data.

The comparison between models trained with and without the county_te feature highlights two important findings:

1. county_te encapsulates essential location-specific information that enhances predictive accuracy; and.
2. even in its absence, substantial predictive capacity remains due to the contributions of climatic and edaphic factors.

Detailed numerical results for all validation protocols are provided in Supplementary Table S7.

## Conclusion

The NSGA-III algorithm, integrated with a metalearner architecture (StackingRegressor), demonstrated a strong capability to generate reliable and interpretable predictions of wheat yield, particularly emphasizing the importance of regional variables such as county_te. By incorporating advanced dimensionality reduction, multi-objective optimization, and explainable AI (XAI) techniques, the proposed framework provided a comprehensive and systematic solution for modeling complex agricultural processes. NSGA-III effectively identified an optimal subset of predictors by reducing data dimensionality and mitigating overfitting, thereby enabling more stable and generalizable model performance.

The selected features collectively highlighted the central role of nonlinear interactions between hyper-climatic variables (e.g., minimum temperature and temperature seasonality) and edaphic–nutritional attributes (e.g., soil salinity, bicarbonate, clay–aluminum interaction, soil organic matter–precipitation interaction, magnesium, and potassium). The inclusion of both quadratic and interaction terms in the final feature set empirically confirms that nonlinear dependencies are fundamental in modeling biological–environmental systems. The final metalearner—combining NSGA-III, LGBM, and DNN—achieved an R^2^ of 0.44 on the test set. Although this value appears moderate, it is reasonable considering the intrinsic complexity and multidimensional structure of the climate–soil–plant system, the wide spatial domain of the study, and the substantial computational workload (including nearly three weeks of repeated model training under diverse hyperparameter settings). As emphasized by Kuhn and Johnson^50^, overreliance on R^2^ can be misleading in real-world machine learning studies; what matters more is the model’s ability to uncover and quantify the underlying processes governing the system—an objective that the present study successfully achieved. Accordingly, the resulting R^2^ values confirm that the selected features capture significant portions of the system’s intricate dependencies and provide a robust foundation for future investigations.

The extensibility of this framework to other high-value crops in Iran is promising. Given the successful modeling of date palm yield using regression and soil inputs^20^, integrating NSGA-III feature selection and SHAP interpretation can enhance precision input management in date production systems, supporting climate-resilient agriculture in arid regions. Similarly, the superior performance of ensemble models such as Random Forest in pistachio yield prediction (R^2^ = 0.96)^21^ suggests that combining NSGA-III-guided feature selection with gradient boosting or stacking frameworks—similar to the current study—may further improve precision and generalizability in perennial cropping systems facing soil degradation and climatic variability. The high accuracy of the SVM–Firefly hybrid on the same soil dataset (94%)^22^ supports the scalability of metaheuristic tuning as a complementary enhancement to our NSGA-III pipeline. Future extensions may include integrating NSGA-III with multi-criteria decision-making approaches such as Fuzzy TOPSIS for optimizing crop allocation under shared soil constraints or coupling NSGA-III with AHP–Matter Element hybrids to unify yield prediction and land-suitability analysis across Iran’s arid agroecosystems.

The results of this study—including detailed evaluation metrics, analytical visualizations, and the fully saved predictive model—offer a practical foundation for designing location-specific management strategies aimed at improving input efficiency and enhancing climate adaptation in wheat production systems. Nevertheless, the observed performance variations across counties, the reduced accuracy in test and validation subsets, and several inherent limitations—including the relatively small dataset, geographic specificity of the study region, sensitivity to NSGA-III hyperparameters (e.g., population size, mutation rate), and influence of unmeasured environmental variables—highlight the need for continued refinement and expansion of the proposed framework.

In addition, two methodological considerations further strengthen the validity of the findings:

- Spatial cross-validation was not required due to the absence of spatial clustering in the data,
- The spatial randomness of residual errors (as confirmed by Moran’s I analysis) increases confidence in the model’s generalizability across counties.

While ecological interpretations (e.g., dominant roles of Tmin and TS) are robust, they should be framed alongside the strong spatial signal captured by county_te, which reflects unobserved local factors. Kling-Gupta Efficiency (KGE) and bootstrapped confidence intervals were not implemented due to computational constraints, though 95% prediction intervals via quantile regression are provided in Supplementary Table S6. The county mean baseline (test R^2^ ≈0.28) further contextualizes the added value of the proposed model.

Altogether, the accompanying figures and tables provide clear and informative visual tools that facilitate the interpretation of these complex interactions, supporting deeper scientific insight and more informed decision-making in future research and agricultural applications.

## Supplementary Information

Supplementary Information 1.

Supplementary Information 2.

Supplementary Information 3.

Supplementary Information 4.

Supplementary Information 5.

Supplementary Information 6.

Supplementary Information 7.

Supplementary Information 8.

## Data Availability

The custom Python code, scripts, processed features, model outputs, and supporting materials developed in this study are openly available in the Zenodo repository at: [10.5281/zenodo.19436213](https:/doi.org/10.5281/zenodo.19436213). The raw dataset cannot be made publicly available due to institutional data sharing policies and ongoing research projects. However, the anonymized processed dataset, selected feature sets, and trained models (.pkl and.h5 files) are available from the corresponding author upon reasonable request.

## Code availability

The custom Python code and supporting materials used in this study, including the hybrid NSGA-III implementation, data preprocessing, feature selection, stacking ensemble modeling, hyperparameter tuning, and visualization scripts, are publicly available at Zenodo with the following 10.5281/zenodo.19436213.
