# Supplementary material for: A novel hybrid NSGA-III and machine learning framework for modeling wheat yield variability using climatic, edaphic, and nutritional drivers
Source: Sci Rep. 2026 May 6;16:20855. doi: 10.1038/s41598-026-48918-0 (PMC13338409; doi:10.1038/s41598-026-48918-0)
Supplement: Supplementary file 1 — Supplementary Information 1. [file 41598_2026_48918_MOESM1_ESM.docx]

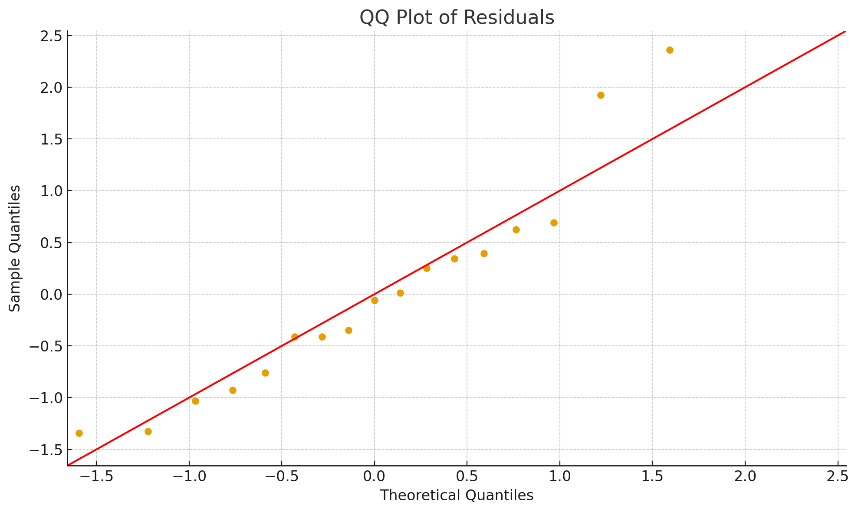


**Figure S2**. **QQ-plot of model residuals (training set)**

Quantile–Quantile plot comparing empirical residual quantiles to theoretical normal quantiles. Deviations from the 45° line at both tails indicate departures from normality (Shapiro–Wilk: W = 0.889, p = 0.033). Although residuals are not perfectly normal, their spatial distribution is random (see Figure 1 and Moran’s I results).
