# Supplementary material for: A novel hybrid NSGA-III and machine learning framework for modeling wheat yield variability using climatic, edaphic, and nutritional drivers
Source: Sci Rep. 2026 May 6;16:20855. doi: 10.1038/s41598-026-48918-0 (PMC13338409; doi:10.1038/s41598-026-48918-0)
Supplement: Supplementary file 2 — Supplementary Information 2. [file 41598_2026_48918_MOESM2_ESM.docx]

**Supplementary Table S1. Sample counts by county and by year (2004–2023)**

**S1-A. Samples per county**

| **County** | **Samples** |
| --- | --- |
| Bardeskan | 19 |
| Chenaran | 19 |
| Daregaz | 19 |
| Fariman | 19 |
| Gonabad | 19 |
| Kalat-e-Nader | 19 |
| Kashmar | 19 |
| Khaf | 19 |
| Mashhad | 19 |
| Neyshabur | 19 |
| Quchan | 19 |
| Roshtkhar | 19 |
| Sabzevar | 19 |
| Sarakhs | 19 |
| Taybad | 19 |
| Torbat-e Heydariyeh | 19 |
| Torbat-e Jam | 19 |
| **Total** | **323** |

**S1-B. Samples per year (Gregorian years)**

| **Year** | **Samples** |
| --- | --- |
| 2004 | 19 |
| 2005 | 19 |
| 2006 | 19 |
| 2007 | 19 |
| 2008 | 19 |
| 2009 | 19 |
| 2010 | 19 |
| 2011 | 19 |
| 2012 | 19 |
| 2013 | 19 |
| 2014 | 19 |
| 2015 | 19 |
| 2016 | 19 |
| 2017 | 19 |
| 2018 | 19 |
| 2019 | 19 |
| 2020 | 19 |
| 2021 | 19 |
| 2022 | 19 |
| **Total** | **323** |
