# Supplementary material for: A novel hybrid NSGA-III and machine learning framework for modeling wheat yield variability using climatic, edaphic, and nutritional drivers
Source: Sci Rep. 2026 May 6;16:20855. doi: 10.1038/s41598-026-48918-0 (PMC13338409; doi:10.1038/s41598-026-48918-0)
Supplement: Supplementary file 3 — Supplementary Information 3. [file 41598_2026_48918_MOESM3_ESM.docx]

**Supplementary Table S2. Percentage of missing values for all variables**

*% missing*

| **Variable** | **Missing (%)** |
| --- | --- |
| All climatic, edaphic, nutritional, and target variables | **0%** |
| Total missing values in the dataset | **0%** |
