# Supplementary material for: A novel hybrid NSGA-III and machine learning framework for modeling wheat yield variability using climatic, edaphic, and nutritional drivers
Source: Sci Rep. 2026 May 6;16:20855. doi: 10.1038/s41598-026-48918-0 (PMC13338409; doi:10.1038/s41598-026-48918-0)
Supplement: Supplementary file 4 — Supplementary Information 4. [file 41598_2026_48918_MOESM4_ESM.docx]

**Supplementary Table S3. Reanalysis datasets used to fill missing climatic variables**

| **Variable type** | **Source used** | **Notes** |
| --- | --- | --- |
| Precipitation | **APHRODITE** | Recommended by Soltani et al. (2025) for Khorasan Razavi Province |
| Temperature (Tmax, Tmin, Tmean) | **AgMERRA** | High-quality agricultural reanalysis. Recommended by Soltani et al. (2025) for Khorasan Razavi Province; Recommended by Farhadi et al., (2024) for North Khorasan Province |
| Temperature (backup source) | **ERA5** | Used when AgMERRA gaps existed. Recommended by Soltani et al. (2025) for Khorasan Razavi Province; Recommended by Farhadi et al., (2024) for North Khorasan Province  Recommended by Soltani et al. (2025) for Khorasan Razavi Province |
