# Supplementary material for: A novel hybrid NSGA-III and machine learning framework for modeling wheat yield variability using climatic, edaphic, and nutritional drivers
Source: Sci Rep. 2026 May 6;16:20855. doi: 10.1038/s41598-026-48918-0 (PMC13338409; doi:10.1038/s41598-026-48918-0)
Supplement: Supplementary file 5 — Supplementary Information 5. [file 41598_2026_48918_MOESM5_ESM.docx]

**Supplementary Table S3 – Measurement Units, Data Sources, and Preprocessing for All Variables**

| **Variable** | **Unit** | **Source / Preprocessing** |
| --- | --- | --- |
| Tmean | °C | Meteorological stations; missing values filled with AgMERRA/ERA5; outliers checked with IQR |
| Tmax | °C | Meteorological stations; same preprocessing |
| Tmin | °C | Meteorological stations; same preprocessing |
| Prec | mm | Meteorological stations; missing values filled with APHRODITE |
| NDO30 | days | Meteorological stations; verified for consistency |
| PGS | mm | Meteorological stations; same as Prec |
| NPGS | days | Meteorological stations; same as NDO30 |
| TmaxGS | °C | Meteorological stations; same preprocessing |
| TminGS | °C | Meteorological stations; same preprocessing |
| TmeanGS | °C | Meteorological stations; same preprocessing |
| AI | – | Calculated from climatic data; unitless |
| ET | mm | Derived from climatic data; scaled for modeling |
| GSL | days | Meteorological stations; calculated from temperature thresholds |
| TS | – | Calculated from temperature seasonality; unitless |
| GDDGS_EC | – | Interaction of GDDGS and EC; computed post preprocessing |
| AI_Clay | – | Interaction of AI and Clay; computed post preprocessing |
| TminGS_N | – | Interaction of TminGS and Nitrogen; computed post preprocessing |
| GDD_SAR | – | Interaction of GDD and SAR; computed post preprocessing |
| Prec_OC | – | Interaction of Precipitation and Organic Carbon; computed post preprocessing |
| Ca+Mg | meq/L | Sum of calcium and magnesium; soil survey; verified |
| Tmean_pH | – | Interaction of Tmin and pH; computed post preprocessing |
| EC | dS/m | Soil survey; outliers verified |
| pH | – | Soil survey; verified |
| SAR | – | Soil survey; verified |
| SO4 | mg/kg | Soil survey; verified |
| HCO3 | meq/L | Soil survey; verified |
| Na | mg/kg | Soil survey; verified |
| Clay | % | Soil survey; verified |
| Silt | % | Soil survey; verified |
| Sand | % | Soil survey; verified |
| CO3 | % | Soil survey; verified |
| OC | % | Soil survey; verified |
| TNV | % | Soil survey; verified |
| SP | % | Soil survey; verified |
| Fe | mg/kg | Soil survey; verified |
| K | mg/kg | Soil survey; verified |
| P | mg/kg | Soil survey; verified |
| N | mg/kg | Soil survey; verified |
| Mg | mg/kg | Soil survey; verified |
| Ca | mg/kg | Soil survey; verified |
| Cl | mg/kg | Soil survey; verified |
| Cu | mg/kg | Soil survey; verified |
| Zn | mg/kg | Soil survey; verified |
| Mn | mg/kg | Soil survey; verified |
