# Supplementary material for: A novel hybrid NSGA-III and machine learning framework for modeling wheat yield variability using climatic, edaphic, and nutritional drivers
Source: Sci Rep. 2026 May 6;16:20855. doi: 10.1038/s41598-026-48918-0 (PMC13338409; doi:10.1038/s41598-026-48918-0)
Supplement: Supplementary file 6 — Supplementary Information 6. [file 41598_2026_48918_MOESM6_ESM.docx]

**Supplementary Table S5. County-level geospatial characteristics and model performance diagnostics used for spatial autocorrelation assessment.**

| County Latitude Longitude Elevation n_samples R² RMSE MAE Willmott_d |
| --- |
| Bardeskan 35.2667 57.9678 1115 12 0.8773 151.01 124.93 0.9637 |
| Chenaran 36.6458 59.1219 1100 13 0.9277 123.54 97.70 0.9780 |
| Daregaz 37.4461 59.1044 1150 14 0.8593 175.31 120.80 0.9522 |
| Fariman 35.7061 59.8500 1400 13 0.7836 253.89 204.23 0.9123 |
| Gonabad 34.3529 58.6836 1100 11 0.6559 328.71 259.95 0.8473 |
| Kalat-E-Nader 36.9950 59.7533 1500 14 0.7342 353.83 309.24 0.9090 |
| Kashmar 35.2383 58.4656 1050 13 0.9345 105.61 80.95 0.9807 |
| Khaf 34.5761 60.1406 1000 14 0.8875 130.76 96.83 0.9674 |
| Mashhad 36.2970 59.6062 985 14 0.7879 297.45 218.13 0.9300 |
| Neyshabur 36.2133 58.7964 1250 13 0.9363 174.05 138.00 0.9810 |
| Quchan 37.1060 58.5090 1350 13 0.9409 173.07 134.33 0.9824 |
| Roshtkhar 34.9744 59.6233 1000 13 0.8013 276.22 220.38 0.9245 |
| Sabzevar 36.2133 57.6770 978 16 0.8440 242.36 161.53 0.9494 |
| Sarakhs 36.5444 61.1572 287 13 0.8722 199.56 151.34 0.9604 |
| Taybad 34.7400 60.7756 845 13 0.8733 239.70 186.36 0.9591 |
| Torbat-E-Jam 35.2444 60.6222 950 13 0.8859 154.16 113.49 0.9638 |
| Torbat-E Heydariyeh 35.2736 59.2197 1450 14 0.8555 264.24 194.99 0.9511 |

This table summarizes the geospatial attributes (latitude, longitude, elevation) and county-level model diagnostics (R², RMSE, MAE, Willmott’s d, sample size) used to examine spatial patterns in prediction errors and to compute Moran’s I. These values correspond to the same counties visualized in Figure 1 (Spatial Residual Error Map).
