# Supplementary material for: A novel hybrid NSGA-III and machine learning framework for modeling wheat yield variability using climatic, edaphic, and nutritional drivers
Source: Sci Rep. 2026 May 6;16:20855. doi: 10.1038/s41598-026-48918-0 (PMC13338409; doi:10.1038/s41598-026-48918-0)
Supplement: Supplementary file 7 — Supplementary Information 7. [file 41598_2026_48918_MOESM7_ESM.docx]

**Supplementary Table S6**. Hyperparameter Settings for Models and Algorithms

| **Algorithm/Model** | **Hyperparameter/Parameter** | **Range (Searched Values)** | **Final Value Used** |
| --- | --- | --- | --- |
| Mutual Information (MI) | random_state | fixed | 42 |
| RFE | n_features_to_select | 3-15 | 15 (or less if fewer features) |
| RFE Estimator (LGBM) | n_estimators | 80-300 | 200 |
|  | learning_rate | 0.01-0.05 | 0.05 |
|  | max_depth | 3-7 | 5 |
|  | min_child_samples | 8-12 | 10 |
|  | random_state | fixed | 42 |
|  | force_col_wise | fixed | True |
| NSGA3 | pop_size | 100-1000 | 200 |
|  | n_gen | 30-300 | 200 |
|  | ref_dirs | fixed | das-dennis with n_partitions=15 |
|  | crossover_prob | 0.80-0.95 | 0.95 |
|  | mutation_prob | 0.2-0.5 | 0.4 |
|  | seed | fixed | 42 |
| Pareto Problem Model (LGBM) | n_estimators | 80-300 | 100 |
|  | learning_rate | 0.01-0.05 | 0.05 |
|  | max_depth | 3-10 | 5 |
|  | min_child_samples | 2-12 | 3 |
|  | num_leaves | 2-15 | 7 |
|  | random_state | fixed | 42 |
|  | force_col_wise | fixed | True |
| Stacking Base Estimator (LGBM) | n_estimators | 50-500 | 200 |
|  | learning_rate | 0.001-0.05 | 0.05 |
|  | max_depth | 3-10 | 5 |
|  | subsample | fixed | 0.8 |
|  | colsample_bytree | fixed | 0.8 |
|  | min_child_samples | 2-12 | 8 |
|  | random_state | fixed | 42 |
|  | force_col_wise | fixed | True |
| Stacking Meta Estimator (DNN) | hidden_layer_sizes | 50-150 | 50 |
|  | learning_rate | 0.001-0.05 | 0.01 |
|  | epochs | 30-120 | 80 |
|  | batch_size | 16-64 | 16 |
|  | dropout_rate | 0.1-0.5 | 0.3 |
|  | L2_lambda | 0.01-0.2 | 0.01 |
|  | verbose | fixed | 0 |
| Stacking Regressor | passthrough | fixed | True |
|  | cv | 3-5 | 5 |
| Grid Search CV | cv (Group KFold n_splits) | 3-5 | 5 |
|  | scoring | fixed | r2 |
| LGBM for SHAP | n_estimators | 300-1000 | 800 |
|  | learning_rate | 0.01-0.05 | 0.03 |
|  | max_depth | fixed | -1 |
|  | subsample | 0.8-1 | 0.9 |
|  | colsample_bytree | 0.8-1 | 0.9 |
|  | min_child_samples | 8-12 | 10 |
|  | random_state | fixed | 42 |
|  | force_col_wise | fixed | True |
