# Supplementary material for: A novel hybrid NSGA-III and machine learning framework for modeling wheat yield variability using climatic, edaphic, and nutritional drivers
Source: Sci Rep. 2026 May 6;16:20855. doi: 10.1038/s41598-026-48918-0 (PMC13338409; doi:10.1038/s41598-026-48918-0)
Supplement: Supplementary file 8 — Supplementary Information 8. [file 41598_2026_48918_MOESM8_ESM.docx]

**Supplementary Table S7**. Model performance under stringent spatial and temporal validation protocols.

| **Protocol** | **R² (with county_te)** | **R² (without county_te)** |
| --- | --- | --- |
| (a) LOCO-CV (leave-one-county-out) | 0.401 | 0.334 |
| (b) Time-blocked CV (train 2004–2018, test 2019–2023) | 0.388 | 0.351 |
| (c) LOCO + Time-blocked | 0.372 | 0.319 |
